# Safety classification of herbal medicine use among hypertensive patients: a systematic review and meta-analysis

**DOI:** 10.3389/fphar.2024.1321523

**Published:** 2024-05-31

**Authors:** Dain Choi, Hyea Bin Im, Soo Jeung Choi, Dongwoon Han

**Affiliations:** ^1^ Department of Global Health and Development, Graduate School, Hanyang University, Seoul, Republic of Korea; ^2^ Institute of Health Services Management, Hanyang University, Seoul, Republic of Korea; ^3^ Department of Preventive Medicine, College of Medicine, Hanyang University, Seoul, Republic of Korea

**Keywords:** hypertension, herbal medicine, safety, adverse effects, systematic review, meta-analysis

## Abstract

**Background:**

The use of herbal medicines (HMs) for the treatment of hypertension (HTN) is increasing globally, but research on the potential adverse effects and safety of HMs in HTN patients is limited. Therefore, this systematic review and meta-analysis aim to determine the global prevalence of HM usage among HTN patients and assess the safety of identified herbs based on current scientific evidence.

**Methods:**

The PubMed/MEDLINE, EMBASE (Ovid), and Cumulated Index to Nursing and Allied Health Literature (CINAHL) databases were searched for cross-sectional studies on the use of HM among HTN patients. Our review includes studies published in English up to the year 2023. After extracting and appraising the data from the studies, a meta-analysis was conducted using the Stata version 16.0 to estimate the pooled prevalence of HM use in patients with HTN (PROSPERO: CRD42023405537). The safety classification of the identified HM was done based on the existing scientific literature.

**Results:**

This study analyzed 37 cross-sectional studies from 21 countries and found that 37.8% of HTN patients used HM to manage their health. The prevalence of HM use varied significantly based on publication year and geographical region. Among the 71 identified herbs, *Allium sativum* L., *Hibiscus sabdariffa* L., and *Olea europaea* L. were the most commonly used. However, four herbs were identified as contraindicated, 50 herbs required caution, and only 11 herbs were considered safe for use.

**Conclusion:**

The study highlights the potential risks of toxicities and adverse effects associated with HM use in the treatment of HTN. Ensuring patient safety involves using safe HMs in appropriate doses and avoiding contraindicated HMs. Future research should focus on identifying commonly used herbs, especially in resource-limited countries with poor HTN management, and additional clinical research is required to assess the toxicity and safety of commonly used HMs.

## 1 Introduction

High blood pressure is a considerable global health concern (Kearney et al., 2005; Mills et al., 2016), presenting a significant risk factor for cardiovascular disease and premature death (Mills et al., 2020; Roth et al., 2020; Koya et al., 2023). Despite the availability of effective treatment options, over half of the diagnosed patients continue to struggle with managing hypertension (HTN) (Mohsen Ibrahim, 2018; Anand et al., 2019; Burnier and Egan, 2019; Mills et al., 2020; Schutte et al., 2021; Zhou et al., 2021), primarily due to poor adherence to antihypertensive medication, adding to the global disease burden (Kearney et al., 2005; Mills et al., 2020; Mohammed Nawi et al., 2021; Schutte et al., 2021; Hamrahian et al., 2022). Several behavioral risk factors are associated with non-adherence to medication (Burnier and Egan, 2019); complementary and alternative medicine (CAM) use is believed to be one of the contributing factors (Krousel-Wood et al., 2004; Krousel-Wood et al., 2010; Dhar et al., 2017). Among different types of CAM, herbal medicine (HM) is the most popular treatment used by HTN patients (Ali-Shtayeh et al., 2013; James et al., 2018b; Kifle et al., 2021; Palileo-Villanueva et al., 2022). HM has gained popularity as a treatment option for HTN, driven by personal beliefs, preference for natural remedies, cultural traditions, and barriers to accessing conventional care (Liwa et al., 2014; Rahmawati and Bajorek, 2017; Azizah et al., 2021).

However, concerns have been raised about the adulteration or contamination of HM with toxic substances such as heavy metals, as well as the risk of adverse effects when HM is taken with conventional medications due to the pharmacological properties of herbs that may interact with antihypertensive drugs (Pathak and Kothiyal, 2013; Posadzki et al., 2013; Ekor, 2014; Anwar et al., 2016; Azizah et al., 2021; Luo et al., 2021; Im et al., 2023). For instance, concurrent consumption of *Azadirachta indica* A. Juss, *Aloe vera* (L.) Burm. f., and *Hibiscus sabdariffa* L., along with antihypertensive drugs, can compromise the clinical effectiveness of conventional medication by reducing drug absorption (Rahmawati and Bajorek, 2017; Azizah et al., 2021). Furthermore, concurrent use increases the risk of adverse drug reactions, including headaches, gastrointestinal disorders, diarrhea, skin reactions, and frequent urination (Amira and Okubadejo, 2007; Olisa and Oyelola, 2009). Despite the lack of scientific evidence supporting the safety and clinical efficacy of HM (Shafiq et al., 2003; Amira and Okubadejo, 2007; Osamor and Owumi, 2010; Tabassum and Ahmad, 2011; Liwa et al., 2014; Asfaw Erku and Basazn Mekuria, 2016), its usage remains high among HTN patients, with as many as 70% using medicinal herbs due to accessibility and affordability, especially among vulnerable groups (McMahon et al., 1973; Clement et al., 2007; Cuzzolin and Benoni, 2009; Nuwaha and Musinguzi, 2013; Owusu et al., 2020).

These issues have raised concerns about the inappropriate use of HM among HTN patients, highlighting the importance of identifying potential toxicity and adverse effects associated with HM use and developing evidence-based clinical guidelines (Burnier and Egan, 2019). However, limited evidence is available to examine the use of HM among HTN patients and to evaluate the safety of commonly used HMs (Xiong et al., 2015a; Azizah et al., 2021). Therefore, this systematic review and meta-analysis aimed to investigate the pooled prevalence of HM use among HTN patients globally and assess the safety of identified herbs based on current scientific evidence.

## 2 Materials and methods

This systematic review was reported to comply with the Preferred Reporting Items for Systematic Reviews and Meta- Analyses (PRISMA) checklist (Moher et al., 2009; Page et al., 2021; Supplementary Table S1). The rationale and methods of the study protocol were registered in the International Prospective Register of Systematic Reviews (PROSPERO, registration number: CRD42023405537).

### 2.1 Search strategy

An electronic database search was conducted on 19 June 2023 and included the systematic investigation of the PubMed/MEDLINE, EMBASE (Ovid), and Cumulated Index to Nursing and Allied Health Literature (CINAHL) databases. The initial version was developed using keywords suggested by the literature based on previous studies, and a comprehensive search strategy including specific words, phrases, and controlled vocabulary was then developed by an information specialist in collaboration with two cardiologists and three public health specialists. The search strategy and results are provided in Supplementary Table S2. Medical subject heading (MeSH) terms, EMBASE subject headings (Emtree), and keywords from related articles were explored to guide the selection of relevant search terms. The search terms were further refined by referring to related literature reviews. Finally, variations in three major terms (hypertension, herbal medicine, and cross-sectional study) were used for the search.

### 2.2 Eligibility criteria

This review includes studies that (i) were published from inception to 2023, (ii) were published in English, (iii) report cross-sectional data of HM use among HTN patients, and (iv) report the name of each herb used and the corresponding number of users. Additionally, studies with mixed study populations (i.e., studies on chronic disease patients that include more than one disease group) were only included if the findings related to the HTN population were presented independently.

Studies were excluded if they met one or more of the following criteria: (i) lack of full English text; (ii) non-use of cross-sectional study designs; (iii) inclusion of non-hypertensive study samples or failure to separate data on hypertensive subjects from other study populations; (iv) failure to report the type of HM used by HTN patients and provide information on the number of users per HM type; (v) incorrect publication types, such as posters, letters, conference abstracts, review articles, or case reports (Mills and Bone, 2004).

### 2.3 Safety classification of identified herbal medicines

The safety of identified herbs was classified into four categories: potentially harmful to use, use with caution, safety evidence not available, and safe to use (Table 1), and these categories were determined based on the previous literature (Kennedy et al., 2016; Ahmed et al., 2017; Kim H.-L. et al., 2023; Im et al., 2023). The safety classification of identified HMs was determined by reviewing the existing scientific literature and reference material, including the latest published literature, websites, and textbooks related to safety (Ulbricht and Basch, 2005; Johnston, 2006; Gruenwald et al., 2007; Ulbricht, 2010; Gardner and McGuffin, 2013), and the quality of the evidence was assessed based on the hierarchy of evidence (Concato et al., 2000). Evidence from clinical studies on HTN patients was considered first, followed by human studies and animal studies.

**TABLE 1:** Safety classification of identified herbal medicines used by HTN patients.

Category	Classification	Description
✕	Contraindicated for use	The available evidence has shown adverse impacts on hypertension, following the use of the herb
△	Should be used with caution	Caution must be taken when using this herb due to the lack of sufficient human evidence or limited research available. Therefore, it is advisable to use this herb under the guidance and supervision of a qualified healthcare practitioner
ᅳ	Safety evidence not available	No reference was found regarding the use of the herb for hypertension
○	Safe to use	Available human evidence suggests that the herb can be safely used by hypertensive patients

If safety information for an HM was not identified in the above reference sources, we conducted an additional search on PubMed, EMBASE, and Google Scholar. When inconsistencies were found among the reviewed sources, we prioritized the most recently published study on the safety classification of an HM as the primary reference source (Ahmed et al., 2017; Im et al., 2023). Lastly, because the current study is primarily concerned with the safety classification of the identified herbs, the efficacy of each herb was simply categorized based solely on whether there was evidence of blood pressure (BP) lowering from either animal or clinical studies.

### 2.4 Data extraction and study quality assessment

Based on the eligibility criteria, three researchers (DC, HI, and SJ) conducted a full-text review using a review template that was developed to examine study characteristics (i.e., publication year, country, study design, setting, and research subjects) and the inclusion of primary study outcomes (i.e., the prevalence of HM use, as well as the type of HM used to treat HTN). The data extracted from each study were compared, and any discrepancies between the three reviewers were resolved through consultation with the senior researcher (DW).

The quality of the included studies was independently evaluated by three reviewers using a validated tool to assess the risk of bias in prevalence studies (Hoy et al., 2012). The tool consists of 10 questions that address four items of external validity of the study and six domains of internal validity issues. A score of 0 (no) or 1 (yes) was given for each item, and scores were summed across items to calculate an overall score that ranged from 0 to 10. Studies were then classified as having a low (0–3), moderate (4–6), or high (7–9) risk of bias.

### 2.5 Data synthesis and statistical analysis

A meta-analysis was conducted to estimate the pooled prevalence of HM use among HTN patients and the corresponding 95% confidence intervals (CI). Articles that did not report the prevalence of HM use (i.e., studies reporting the prevalence of CAM, biologically based therapies, or home remedy use) were excluded from the meta-analysis. The prevalence of HM use from each study was initially recorded in the Microsoft Excel spreadsheet. If a study only reported the number or percent values of HTN patients or HM use, the researchers re-calculated the value of events based on the given percentages. The recorded values were then imported into the Stata version 16.0 tool for further analysis. Due to high heterogeneity among the studies (I^2^ = 99.61%), a random-effect meta-analysis was conducted. The results were displayed using a forest plot.

The presence of publication bias was evaluated using the funnel plot and the Egger test (*p* = 0.013; Supplementary Figure S3). A subgroup meta-analysis was conducted to investigate potential differences in the use of HM by geographical region (i.e., individual countries and continents) and the publication year (i.e., studies published before 2011 *versus* after 2011).

## 3 Results

### 3.1 Selection of studies

**FIGURE 1:**
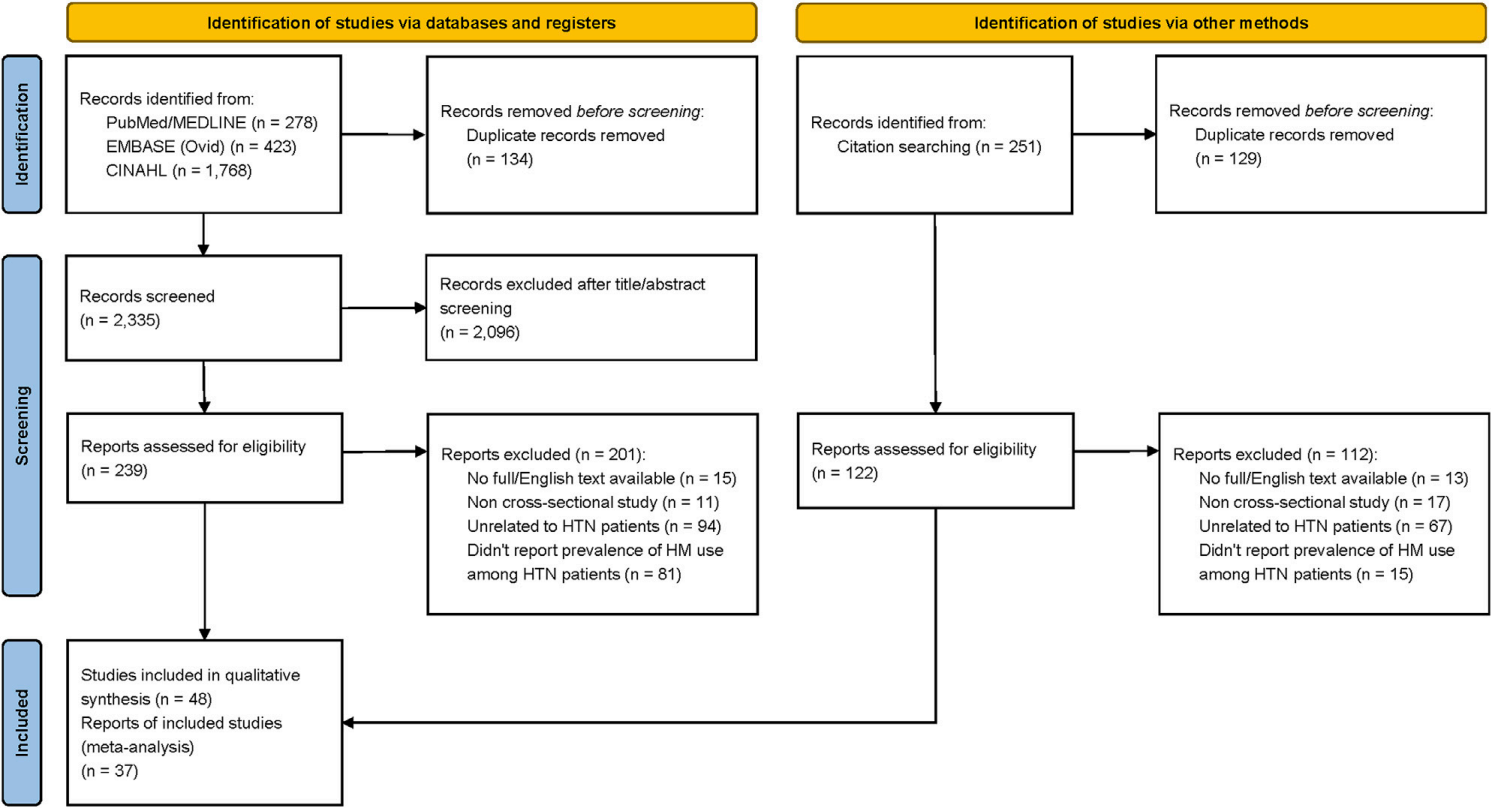
Flow diagram of the search strategy.

The PRISMA flow diagram of the study selection process is shown in Figure 1. A total of 2,469 studies were identified from databases and other sources. After removing duplicate records, 2,335 articles were eligible for title and abstract review. During the title and abstract screening, 2,096 records were excluded, leaving 239 articles that were selected for the full-text review. During the full-text review, 201 articles were excluded for the following reasons: unavailability of full-text or English text, ineligible study design or publication type (i.e., reviews, conference abstracts, the letter to the editor, posters, case reports, and animal studies), unrelated to HTN patients, and insufficient reporting of the prevalence of HM use by HTN patients.

A total of 251 additional records were identified by reviewing the reference lists of the 38 included studies. After removing duplicates, 122 additional studies were included for further review. However, during the full-text appraisal, 112 studies were excluded as they did not meet the inclusion criteria (i.e., unrelated to HTN patients, non-cross-sectional study). As a result, 48 articles were eligible for the risk of bias assessment.

### 3.2 Study quality and risk of bias assessment

The external validity of the reviewed studies showed a high risk of bias for the target population (item 1), sampling frame (item 2), and random sample selection (item 3) because most studies included in this review were conducted at a single hospital and used the convenience sampling method. In addition, as for the internal validity, most items exhibited a low risk of bias, except for item 9. The prevalence period (item 9) was considered to have a high risk of bias if a study did not report or examine the respondent’s HM use beyond the past 12 months. As a result, out of the 48 studies examined, 11 showed a high risk of bias, 7 exhibited a low risk of bias, and 30 displayed a moderate risk. Therefore, excluding 11 studies with a high risk of bias, 37 studies were included in the final review (Supplementary Table S4).

### 3.3 Characteristics of cross-sectional studies of HM use among HTN patients

In the review, a total of 37 cross-sectional studies conducted in 21 countries were examined. Among these, 27 studies were published after 2010, while ten were conducted between 2000 and 2010. The characteristics of the studies included in the review are illustrated in Table 2. The highest number of studies were carried out in Asia (48.6%), followed by Africa (35.1%), North America (13.5%), and Europe (2.7%). The sample sizes varied from a minimum of 19 participants to a maximum of 2,436 participants (Mahfudz and Chan, 2005; Ali-Shtayeh et al., 2013). Fifteen studies provided detailed information regarding the specific types of HM used and the number of users for each herb (Amira and Okubadejo, 2007; Clement et al., 2007; Gohar et al., 2008; Olisa and Oyelola, 2009; Ali-Shtayeh et al., 2013; Bahar et al., 2013; Kretchy et al., 2014; Tajadini et al., 2015; Baran et al., 2017; Liwa et al., 2017; James et al., 2018a; Al-Hadid et al., 2020; Adeniyi et al., 2021; Joachimdass et al., 2021; Kifle et al., 2021).

**TABLE 2:** Characteristics of included studies.

No.	Study (author, year)	Country	Setting	Population	Sample size^a^ (Response rate)	HM users N (%)	No. of herbs identified^e^
Total					23,947^*^	6,316	166^d^
1	Shafiq et al. (2003)	India	HTN clinic of a teaching hospital	HTN patients visiting the HTN clinic	333 (63.9%)	48 (14.4%)	NR
2	Mahfudz and Chan (2005)	Malaysia	Public primary care center	HTN patients attending the outpatient HTN department	124 (NR)	19 (15.3%)	6
3	Yeh et al. (2006)	United States	National household survey (National Health Interview Survey)	Adults ≥18 years of age	Total: 31,044 (NR)	65 (0.8%)	NR
					HTN: 8,055^*^		
4	Amira and Okubadejo (2007)	Nigeria	HTN clinic of the university teaching hospital	HTN patients attending the HTN clinic for at least 6 months	225 (NR)	88 (39.1)^b^	5
5	Clement et al. (2007)	Trinidad and Tobago	16 primary healthcare facilities	Patients ≥16 years of age who confirmed their use of herbal remedies	Total: 265 (NR)^c^	53 (71.6%)	15
					HTN: 74^*^ (28.0%)		
6	Gohar et al. (2008)	United Kingdom	Secondary teaching hospital	HTN patients attending the outpatient HTN clinic	153 (78.1%)	24 (15.7%)^c^	4
7	Olisa and Oyelola (2009)	Nigeria	Secondary hospital (state level)	Ambulatory HTN patients attending the HTN clinic	480 (96.0%)	120 (25.0%)	24
8	Al-Hamdan et al. (2010)	Saudi Arabia	Community-based survey in 20 primary health centers	All Saudi population aged 15–64 years	Total: 4,719 (99.2%)	45 (8.3%)	NR
					HTN: 542^*^		
9	Delgoda et al. (2010)	Jamaica	Eighteen pharmacies	Patients or parents/carers of children visiting the study pharmacy	365 (91.5%)	103 (79.8%)	NR
10	Nur (2010)	Turkey	Community-based survey at a semi-rural province	Adults ≥18 years of age	3,876 (96.3%)	273 (48.8%)	NR
					HTN: 559^*^		
11	Ali-Shtayeh et al. (2013)	Palestine	HTN outpatient departments at governmental hospitals, military medical clinics, and refugee camp clinics in eight towns	HTN patients who had been diagnosed with HTN and attending the HTN outpatient clinic	4,575 (NR)	2,436 (53.25%)^b^	83
12	Bahar et al. (2013)	Turkey	Three primary care centers located within the same district	Patients who had been diagnosed with HTN by a physician, receiving HTN treatment, and admitted to a primary care center	193 (NR)	99 (51.3%)	8
13	Wazaify et al. (2013)	Jordan	University teaching hospital	Patients with CKD, dyslipidemia, and HTN cases attending the outpatient departments	Total: 700 (91.3%)	44 (6.9%)^c^	9
					HTN: 636^*^		
14	Nuwaha and Musinguzi (2013)	Uganda	Community-based survey at two rural districts	HTN patients ≥15 years of age	258 (91.8%)	73 (28.3%)	NR
15	Hu et al. (2018)	China	Survey conducted within a local community of a metropolitan city	HTN patients ≥35 years of age who have had HTN for a minimum of 12 months	318 (81.4%)	59 (18.6%)	NR
16	Mollaoğlu et al. (2013)	Turkey	Outpatient clinics of a general hospital	Adults ≥21 years of age and treated for one or more of six chronic diseases, including HTN, within the past year in Turkey	252 (NR)	51 (61.4%)	NR
					HTN: 83^*^		
17	Açıkgöz et al. (2014)	Turkey	Tertiary care education hospital	All patients admitted to outpatient cardiology clinics with prior prescription of at least one cardiovascular drug	390 (84.5%)	79 (29.7%)	NR
					HTN: 266^*^		
18	Kretchy et al. (2014)	Ghana	Two tertiary teaching Hospitals	HTN patients ≥18 years of age attending the outpatient departments	400 (100.0%)	51 (12.8%)	13
19	Boima et al. (2015)	Ghana and Nigeria	Three tertiary teaching hospitals and one general hospital	HTN patients ≥18 years of age who had been diagnosed with HTN and placed on medication for at least 12 months	357 (NR)	62 (17.4%)	NR
20	Li et al. (2015)	China	Community-based survey at the two rural counties	HTN patients ≥30 years of age in two counties	665 (NR)	93 (14.0%)	NR
21	Tajadini et al. (2015)	Iran	Telephone interview	HTN patients participated in the KERCADER project in the Beast subspecialty clinic	612 (94.2%)	180 (29.4%)	3
22	Asfaw Erku and Basazn Mekuria (2016)	Ethiopia	University teaching hospital	HTN patients ≥18 years of age who started taking medication for reduction of BP and visited the outpatient clinic	412 (97.4%)	189 (45.9%)	NR
23	Lulebo et al. (2017)	Congo	Fifteen primary healthcare facilities	HTN patients >18 years of age attending Kinshasa Primary Healthcare (KPHC) facilities	280 (NR)	119 (42.5%)	NR
24	Baran et al. (2017)	Turkey	Tertiary hospital	HTN patients ≥18 years attending the Family Health Center	465 (80.4%)	259 (55.7%)^b^	2
25	Liwa et al. (2017)	Tanzania	Tertiary teaching hospital	Patients >18 years of age admitted with HTN-related diagnoses	213 (92.6%)	52 (24.4%)	15
26	Adidja et al. (2018)	Cameroon	Community-based survey at a single district	HTN patients >21 years of age who were on hypertensive medication(s) for at least 1 month	183 (NR)	38 (20.8%)	NR
27	James et al. (2018a)	Sierra Leone	Four public and two private health facilities	HTN patients ≥18 years of age attending the outpatient departments	260 (NR)	148 (56.9%)	14
28	Peltzer& Pengpid (2019)	Thailand	Seven district hospitals	Outpatients ≥21 years of age and had a chronic disease	1,396 (98.6%)	272 (32.5%)	NR
					HTN: 838^*^		
29	Sabery et al. (2019)	Iran	Community-based survey at Kashan city	Adults >60 years of age	770 (100.0%)	235 (68.5%)	NR
					HTN: 343^*^		
30	Al-Hadid et al. (2020)	Jordan	A health center	HTN patients ≥16 years of age who had been managed at a selected health center for at least 6 months	208 (100.0%)	107 (51.4%)	4
31	Alshabi (2020)	Saudi Arabia	University hospital	Adults >18 years of age	1,000 (NR)	51 (43.6%)	NR
					HTN: 117^*^		
32	El-Dahiyat et al. (2020)	Jordan	Two universities	University students, staff, and their family members	378 (75.6%)	32 (88.9%)	NR
					HTN: 36^*^		
33	Owusu et al. (2020)	Jamaica	Any of the seven chronic disease clinics in one of the four parishes under the WRHA	Adults ≥18 years of age who had been diagnosed with HTN and/or T2DM and were attending a chronic disease clinic	Total: 362 (95.3%)	224 (72.1%)^a^	6
					HTN: 311^*^ (90.1%)		
34	Adeniyi et al. (2021)	Jamaica	Clinics for HTN and T2DM in the four parishes	Adults ≥18 years of age who had been diagnosed with HTN and/or T2DM and attended health clinics in one of the four WRHA parishes	60 (NR)	48 (96.0%)	6
					HTN: 50^*^		
35	Joachimdass et al. (2021)	Malaysia	Primary healthcare clinic in the suburban district	HTN patients ≥18 years of age who had attended the clinic for at least three prior appointments for HTN	294 (96.1%)	90 (30.6%)	52
36	Kifle et al. (2021)	Ethiopia	General hospital located in a town	HTN patients ≥18 years of age who received medical care at the adult hypertensive care services	450 (94.7%)	167 (37.1%)	9
37	Thangsuk et al. (2021)	Thailand	Primary care clinic in university hospital	Patients ≥35 years of age who had been diagnosed with essential HTN and taking at least one antihypertensive drug	450 (80.6%)	80 (17.8%)	NR

HTN, hypertension; T2DM, Type 2 diabetes mellitus; CKD, chronic kidney disease; BP, blood pressure; HL, hyperlipidemia; HM, herbal medicine; CAM, complementary and alternative medicine; NR, not reported.

* The original study examined the use of HM among both HTN patients and non-HTN adults; thus, only the data on HTN patients were extracted and included in this review.

^a^ The researchers manually calculated the exact number of respondents as the prevalence was only reported as percentages in the published article.

^b^ This number indicates the number of CAM users as the original study encompasses the use of CAM, and only the number of individual HM users is reported (the overall number of HM users is not provided).

^c^ The original study encompasses the use of complementary medicine; thus, only the data on HM use were extracted and included in this review.

^d^ The total number of HMs indicated in this table excludes duplicate records (e.g., if an HM mortality was reported in more than one study, it is counted once).

^e^ The total number of herbal medicines with their full names mentioned in each study.

### 3.4 Prevalence of HM use among HTN patients

**FIGURE 2:**
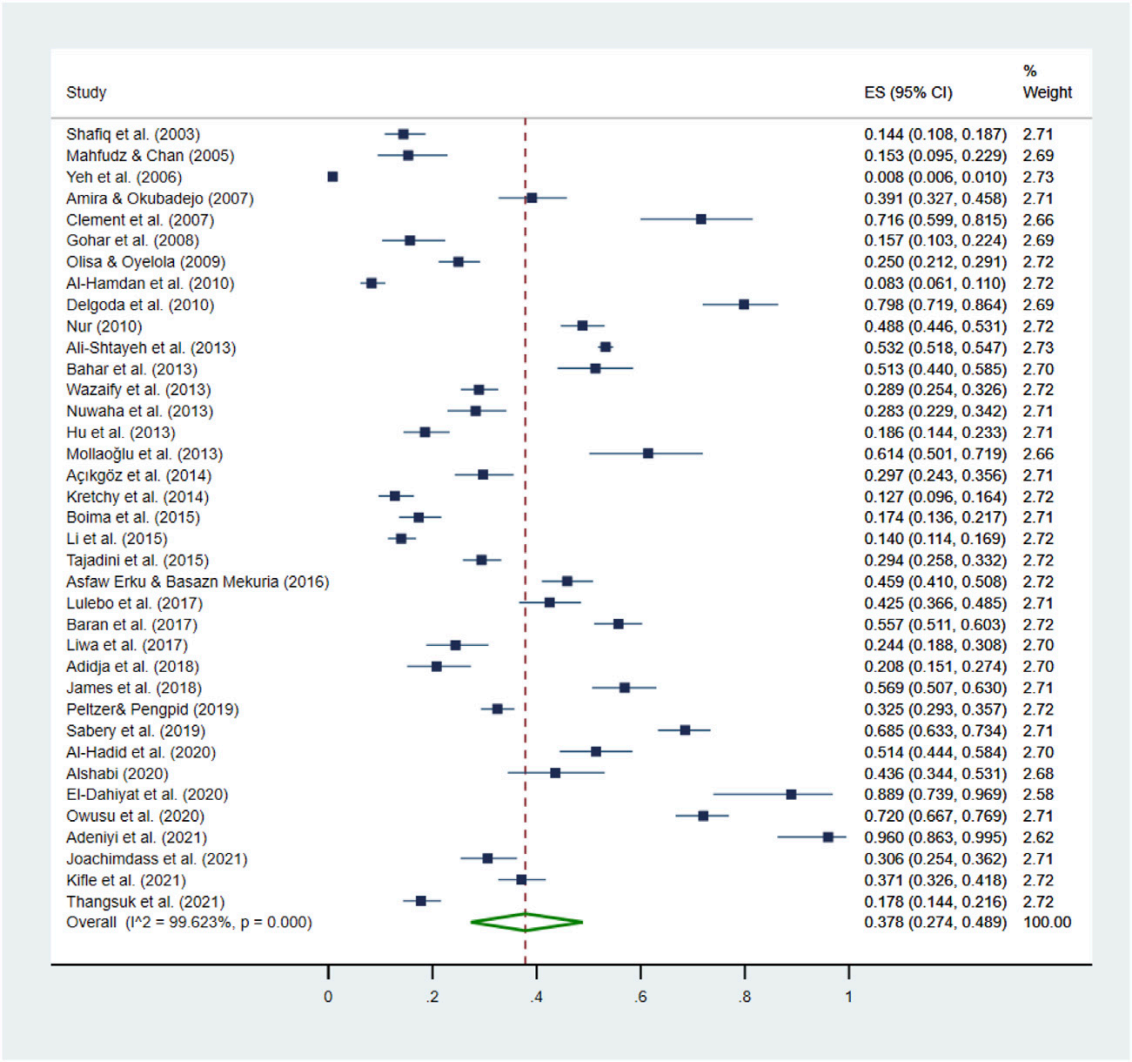
Pooled prevalence of HM use by HTN patients. This figure presents a forest plot of a random-effect meta-analysis. Thirty-seven studies on HTN patients reported the HM use rate and were included in the pooled estimation of HM use. The square blue dots and the dashed line passing through represent the effect size and corresponding 95% confidence intervals (CIs) reported in individual studies, and the green diamond on the bottom and the size of its lateral tips denote the pooled effect size and its 95% CI.

**FIGURE 3:**
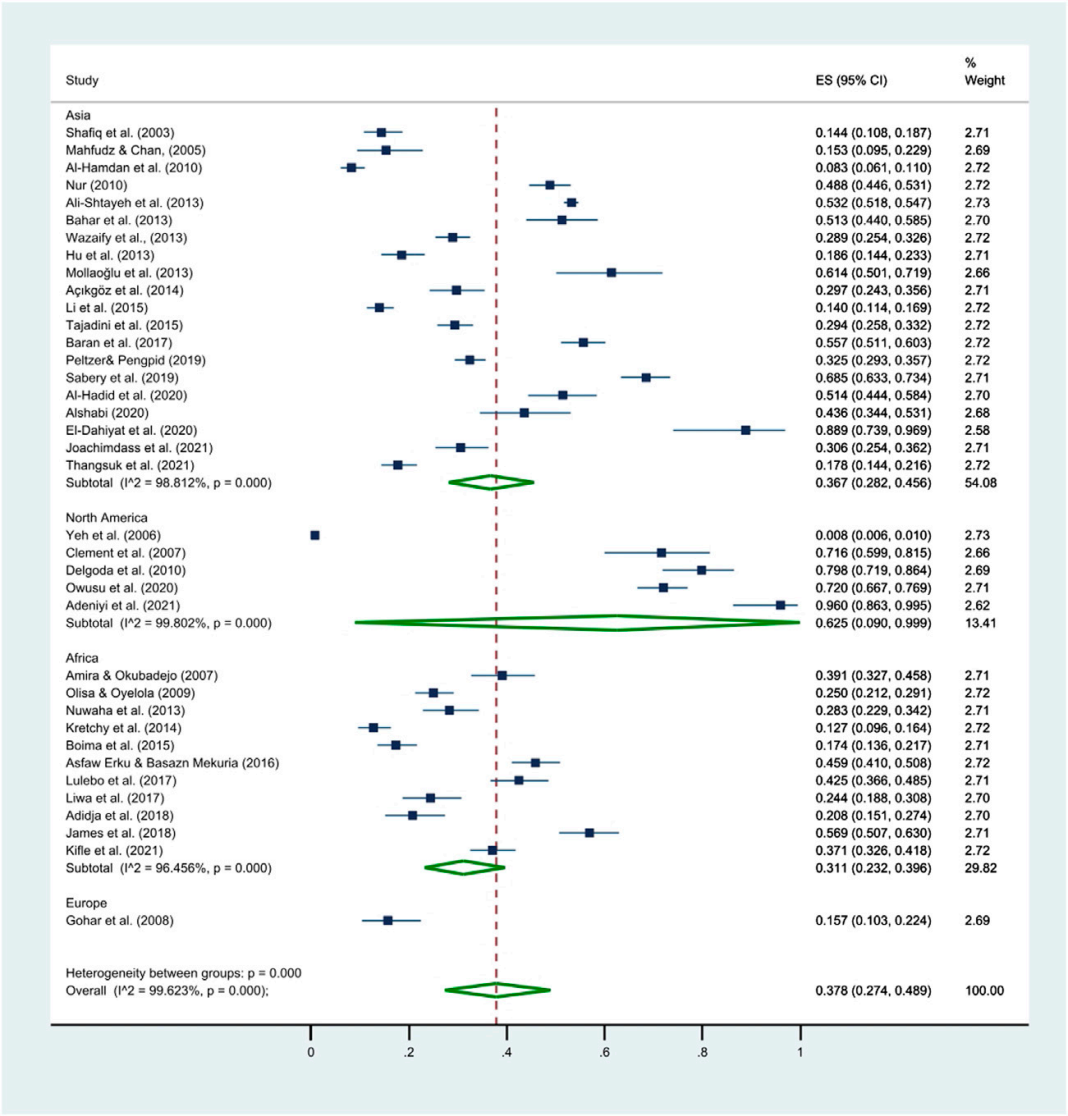
Differences in the pooled prevalence of HM use by continents. This figure presents a forest plot of a random-effect meta-analysis. Subgroup meta-analysis was conducted to investigate potential differences in the prevalence of HM use by geographical region (i.e., Asia, North America, Africa, and Europe). The square blue dots and dashed line passing through represent the effect size and corresponding 95% CI reported in individual studies, and the green diamond on the bottom and the size of its lateral tips denote the pooled effect size and its 95% CI for each subgroup.

The prevalence of HM use among HTN patients ranged from 0.8% to 96.0% (Yeh et al., 2006; Adeniyi et al., 2021), the pooled prevalence was 37.8% (95% CI: 27.4%–48.9%; Table 3; Figure 2). Among the 21 countries included in this review, HM use was the lowest in the United States and the highest in Jamaica (Wazaify et al., 2013; Owusu et al., 2020). The prevalence of HM utilization varied significantly by publication year and geographic region, with higher utilization rates observed in studies published after 2011 (41.0%, 95% CI: 33.9%–48.3%) than those published before 2011 (28.6%, 95% CI: 11.3%–50.1%; *p* < 0.001). The highest use was reported in North America (62.5%, 95% CI: 9.0%–99.9%), followed by Asia (36.7%, 95% CI: 28.2%–45.7%) and Africa (30.9%, 95% CI: 22.8%–39.6%; Table 3; Figure 3).

**TABLE 3:** Pooled prevalence of HM use by study characteristics.

Characteristic	Included studies^a^	Sample size	Mean^b^ (%)	95% CI	*p*-value
Overall	37	23,947	37.8	27.4–48.9	
Publication year					
Before 2011	10	10,674	28.6	11.3–50.1	<0.001
After 2011	27	13,273	41.0	33.9–48.3	
Geographical region					
Africa	11	3,518	30.9	22.8–39.6	<0.001
Asia	20	11,657	36.7	28.2–45.7	
Europe	1	153	15.7	10.3–22.4	
North America	5	8,619	62.5	9.0–99.9	

*Estimated using a random-effects model.

^a^Number of studies included in each subgroup.

^b^Pooled estimate.

### 3.5 The most commonly used HM and reported indications for HM use

The use of 165 herbs was observed in the reviewed articles, but only the modalities used by ten or more study subjects were included in the safety evaluation. As a result, the use of 71 different HMs (individual herbs or mixture as preparation) was identified from 15 studies (Table 4). The most frequently used herbal medicines included *Allium sativum* L. (31.3%), *H. sabdariffa* L. (12.3%), *Olea europaea* L. (10.4%), and *Crataegus oxyacantha* L (8.0%).

**TABLE 4:** Most commonly used HMs and reported indications for HM use among HTN patients.

No.	Herbal medicines^b^		No. of users^a^ (Total= 3,922) N (%)	Route	Reported indication for HM use
	English name	Scientific name			
1	Garlic^a–o^	*Allium sativum* L.	1,229 (31.3)	Oral, topical, inhalation	Control or treat HTN, DM, or cancerreducing the side effects of prescription drugs; carminative; improve overall health; malaria; weight reduction; dyslipidemia; arthritis; hyperlipidemia; and meningitis
2	Roselle^f^ ^,^ ^m^	*Hibiscus sabdariffa* L.	484 (12.3)	Oral, topical, and inhalation	Control or treat HTN or DM
3	Olive^f^	*Olea europaea* L.	407 (10.4)	Oral, topical, and inhalation	Control or treat HTN, DM, or cancer
4	Hawthorn^f^	*Crataegus oxyacantha* L.	314 (8.0)	Oral, topical, and inhalation	Control or treat HTN, DM, or cancer
5	Lemon^f^ ^,^ ^g^ ^,^ ^j^ ^,^ ^k^ ^,^ ^o^	*Citrus limon* L.	288 (7.3)	Oral	Control or treat HTN, DM, or cancer, reducing the side effects of prescription drugs
6	Green tea^f^ ^,^ ^g^ ^,^ ^i^	*Camellia sinensis* (L.) Kuntze	248 (6.3)	Oral, topical, and inhalation	Control or treat HTN, DM, or cancer
7	Ginger^b^ ^,^ ^e^ ^,^ ^f^ ^,^ ^k^ ^,^ ^n^	*Zingiber officinale* Rosc.	242 (6.2)	Oral and topical	Control or treat HTN, DM, or cancer, reducing the side effects of prescription drugs
8	Anise^f^	*Pimpinella anisum* L.	229 (5.8)	Oral, topical, and inhalation	Control or treat HTN, DM, or cancer, reducing the side effects of prescription drugs
9	Chamomile^f^ ^,^ ^l^	*Matricaria chamomilla* L.	211 (5.4)	Oral, topical, and inhalation	Control or treat HTN, DM, or cancer, reducing the side effects of prescription drugs, and malaria
10	Common sage^f^	*Salvia officinalis* L.	181 (4.6)	Oral, topical, and inhalation	Control or treat HTN, DM, or cancer, reducing the side effects of prescription drugs
11	Rosemary^f^	*Salvia rosmarinus* L.	142 (3.6)	Oral, topical, and inhalation	Control or treat HTN, DM, or cancer, reducing the side effects of prescription drugs
12	Cinnamon^f^ ^,^ ^g^ ^,^ ^m^	*Cinnamomum verum* J. Presl.	121 (3.1)	Oral, topical, and inhalation	Control or treat HTN, DM, or cancer, reducing the side effects of prescription drugs
13	Fenugreek^f^ ^,^ ^o^ ^,^ ^p^	*Trigonella foenum-graecum* L.	118 (3.0)	Oral, topical, and inhalation	Control or treat HTN, DM, or cancer, reducing the side effects of prescription drugs
14	Moringa^e^ ^,^ ^h^ ^,^ ^k^ ^,^ ^l^ ^,^ ^n^ ^,^ ^o^	*Moringa oleifera* L.	118 (3.0)	Oral	Control or treat HTN or DM, weight reduction, arthritis, hyperlipidemia, meningitis, and tuberculosis
15	Peppermint^f^ ^,^ ^p^	*Mentha piperita* L.	117 (3.0)	Oral, topical, and inhalation	Control or treat HTN or cancer, reducing the side effects of prescription drugs
16	Syrian oregano^f^	*Majorana syriaca* (L.) Rafin.	117 (3.0)	Oral, topical, and inhalation	Control or treat HTN, DM, or cancer, reducing the side effects of prescription drugs
17	Parsley^f^	*Petroselinum crispum* (Mill.) Nyman	109 (2.8)	Oral, topical, and inhalation	Control or treat HTN, DM, or cancer, reducing the side effects of prescription drugs
18	African moringa^p^	*Moringa stenoptela* (Baker f.) Cufod.	105 (2.7)	Oral	Control or treat HTN
19	Black cumin^f^	*Nigella sativa* L.	91 (2.3)	Oral, topical	Control or treat HTN, DM, or cancer, reducing the side effects of prescription drugs
20	Honey^k^ ^,^ ^l^	Honey	91 (2.3)	Oral	Control or treat HTN or DM, weight reduction, arthritis, asthma, and meningitis
21	Damakase^p^	*Ocimum lamiifolium* Hochst. ex Benth.	81 (2.1)	Oral	Control or treat HTN
22	Cat thyme^f^	*Teucrium polium* L.	79 (2.0)	Oral, topical, and inhalation	Control or treat HTN, DM, or cancer, reducing the side effects of prescription drugs
23	Chickweed^f^	*Stellaria media* L.	75 (1.9)	Oral and topical	Control or treat HTN, reducing the side effects of prescription drugs
24	Marshmallow^i^	*Althaea officinalis* L.	63 (1.6)	Oral	Control or treat HTN and DM
25	Wild laburnum^p^	*Calpurnia aurea* (Ait.) Benth.	62 (1.6)	Inhalation (nasal)	Control or treat HTN
26	Pomegranate^f^	*Punica granatum* L.	57 (1.5)	Oral and topical	Control or treat HTN, DM, or cancer, reducing the side effects of prescription drugs
27	Guava^f^ ^,^ ^o^	*Psidium guajava* L.	53 (1.4)	Oral, topical, and inhalation	Control or treat HTN, DM, or cancer, reducing the side effects of prescription drugs
28	Banana^f^ ^,^ ^o^	*Musa paradisiaca* L.	48 (1.2)	Oral and topical	Control or treat HTN or cancer, reducing the side effects of prescription drugs
29	Barley^f^	*Hordeum vulgare* L.	45 (1.1)	Oral, topical, and inhalation	Control or treat HTN, DM, or cancer, reducing the side effects of prescription drugs
30	Nepal dock^p^	*Rumex nepalensis* Spreng.	44 (1.1)	Oral	Control or treat HTN
31	Fennel^f^	*Foeniculum vulgare* Mill.	40 (1.0)	Oral, topical, and inhalation	Control or treat HTN, DM, or cancer, reducing the side effects of prescription drugs
32	Onion^f^ ^,^ ^k^	*Allium cepa* L.	40 (1.0)	Oral, topical, and inhalation	Control or treat HTN, DM, or cancer, reducing the side effects of prescription drugs
33	Apple^f^ ^,^ ^g^ ^,^ ^o^	*Malus domestica* Borkh.	30 (0.8)	Oral	Control or treat HTN, DM, or cancer, reducing the side effects of prescription drugs
34	Sweet-marjoram^f^	*Origanum majorana* L.	30 (0.8)	Oral, topical, and inhalation	Control or treat HTN, DM, or cancer, reducing the side effects of prescription drugs
35	Indian pennywort^o^	*Centella asiatica* L.	28 (0.7)	Oral	Control or treat HTN
36	Almond^f^	*Prunus dulcis* L.	25 (0.6)	Oral and topical	Control or treat HTN, DM, or cancer, reducing the side effects of prescription drugs
37	Lupine^f^	*Lupinus albus* L.	25 (0.6)	Oral, topical, and inhalation	Control or treat HTN, DM, or cancer, reducing the side effects of prescription drugs
38	African redwood^p^	*Hagenia abyssinica* J.F. Gmel.	24 (0.6)	Oral	Control or treat HTN
39	Carrot^f^ ^,^ ^k^ ^,^ ^o^	*Daucus carota* L.	24 (0.6)	Oral	Control or treat HTN or cancer, reducing the side effects of prescription drugs
40	Papaya^e^ ^,^ ^k^ ^,^ ^o^	*Carica papaya* L.	24 (0.6)	Oral	Control or treat HTN
41	Sweet peppers^f^	*Capsicum annuum* L.	24 (0.6)	Oral	Control or treat HTN or DM, reducing the side effects of prescription drugs
42	Bitter gourd^o^	*Momordica charantia* L.	23 (0.6)	Oral	Control or treat HTN
43	Black mulberry^f^	*Morus nigra* L.	23 (0.6)	Oral, topical	Control or treat HTN or DM, reducing the side effects of prescription drugs
44	Cucumber^f^ ^,^ ^o^	*Cucumis sativus* L.	22 (0.6)	Oral	Control or treat HTN
45	Safflower^i^	*Carthamus tinctorius* L.	22 (0.6)	Oral	Control or treat HTN and DM
46	Bitter leaf^b^ ^,^ ^h^ ^,^ ^l^ ^,^ ^o^	*Vernonia amygdalina* Delile	21 (0.5)	Oral	Control or treat HTN, improve overall health, weight reduction, arthritis, malaria, typhoid, abdominal pain, and tuberculosis
47	White wormwood^f^	*Artemisia herba-alba* Asso.	21 (0.5)	Oral	Control or treat HTN or DM, reducing the side effects of prescription drugs
48	Palestine arum^f^	*Arum palaestinum* Boiss.	20 (0.5)	Oral and topical	Control or treat HTN or cancer, reducing the side effects of prescription drugs
49	Cabbage^f^ ^,^ ^o^	*Brassica oleracea* L.	19 (0.5)	Oral and topical	Control or treat HTN, DM, or cancer, reducing the side effects of prescription drugs
50	Tosign (Dry thyme)^p^	*Thymus schimperi* R.	19 (0.5)	Oral	Control or treat HTN
51	Licorice^f^	*Glycyrrhiza glabra* L.	16 (0.4)	Oral, topical, and inhalation	Control or treat HTN, reducing the side effects of prescription drugs
52	Pink flax^f^	*Linum pubescens* Willd. ex Schult.	16 (0.4)	Oral and topical	Control or treat HTN or cancer, reducing the side effects of prescription drugs
53	Sugar beet^f^	*Beta vulgaris* L.	16 (0.4)	Oral and topical	Control or treat HTN, reducing the side effects of prescription drugs
54	Mekmeko^f^	*Rumex abyssinicus* Jacq.	15 (0.4)	Oral	Control or treat HTN
55	Wild mustard^f^	*Sinapis arvensis* L.	15 (0.4)	Oral and topical	Control or treat HTN, DM, or cancer, reducing the side effects of prescription drugs
56	Kiwi^f^ ^,^ ^o^	*Actinidia deliciosa* A. Chev	14 (0.4)	Oral	Control or treat HTN, reducing the side effects of prescription drugs
57	Pear^f^ ^,^ ^h^	*Pyrus communis* L.	14 (0.4)	Oral and topical	Control or treat HTN, reducing the side effects of prescription drugs
58	Aloe^b^ ^,^ ^e^ ^,^ ^k^	*Aloe vera* (L.) Burm.f.	13 (0.3)	Oral	Control or treat HTN or malaria
59	Flax seed^g^ ^,^ ^m^	*Linum usitatissimum* L.	13 (0.3)	Oral	Control or treat HTN
60	Lime^n^ ^,^ ^o^	*Citrus aurantiifolia* (Christm.) Swingle	13 (0.3)	Oral	Control or treat HTN
61	Neem^e^	*Azadirachta indica* A. Juss.	13 (0.3)	Oral	Control or treat HTN or malaria
62	Roman nettle^f^	*Urtica pilulifera* L.	13 (0.3)	Oral and topical	Control or treat HTN, DM, or cancer, reducing the side effects of prescription drugs
63	Coffee^f^	*Coffea arabica* L.	12 (0.3)	Inhalation and topical	Control or treat HTN, reducing the side effects of prescription drugs
64	Guinea henweed^n^	*Petiveria alliacea* L.	12 (0.3)	Oral	Control or treat HTN
65	Grape^f^ ^,^ ^o^	*Vitis vinifera* L.	11 (0.3)	Oral	Control or treat HTN or cancer, reducing the side effects of prescription drugs
66	Persimmon^f^	*Diospyros kaki* L.	11 (0.3)	Oral	Control or treat HTN, reducing the side effects of prescription drugs
67	Petai^o^	*Parkia speciosa* Hassk.	10 (0.3)	Oral	Control or treat HTN
68	Prickly pear^f^	*Opuntia ficus-indica* (L.) Mill.	10 (0.3)	Oral and topical	control or treat HTN or cancer, reducing the side effects of prescription drugs
69	Sesame^f^	*Sesamum indicum* L.	10 (0.3)	Oral and topical	Control or treat HTN, DM, or cancer, reducing the side effects of prescription drugs
70	Tamarind^e^ ^,^ ^o^	*Tamarindus indica* L.	10 (0.3)	Oral	Control or treat HTN
71	White micromeria^f^	*Micromeria fruticosa* (L.) Druce	10 (0.3)	Oral and topical	Control or treat HTN

HTN, hypertension; T2DM, Type 2 diabetes mellitus; HM, herbal medicine.

^a^ Table 4 includes studies that provide the specific names of the HMs used by HTN patients, along with the corresponding number of users exceeding 10.

^b^ Superscript numbers from 1 to 15 on every herbal modality indicate the study that reported use of that modality: Amira et al.

^c^ Clement et al.

^d^ Gohar et al.

^e^ Olisa et al.

^f^ Ali-Shtayeh et al.

^g^ Bahar et al.

^h^ Kretchy et al.

^i^ Tajadini et al.

^j^ Baran et al.

^k^ Liwa et al.

^l^ James et al.

^m^ Al-Hadid et al.

^n^ Adeniyi et al.

^o^ Joachimdass et al.

^p^Kifle et al.

The most commonly reported indications for HM use were to control or treat HTN and diabetes mellitus-related symptoms, followed by weight reduction, arthritis, meningitis, hyperlipidemia, malaria, and tuberculosis (Table 4). Oral administration of the HM was the most common route of administration.

### 3.6 Safety classification of commonly used HMs

**FIGURE 4:**
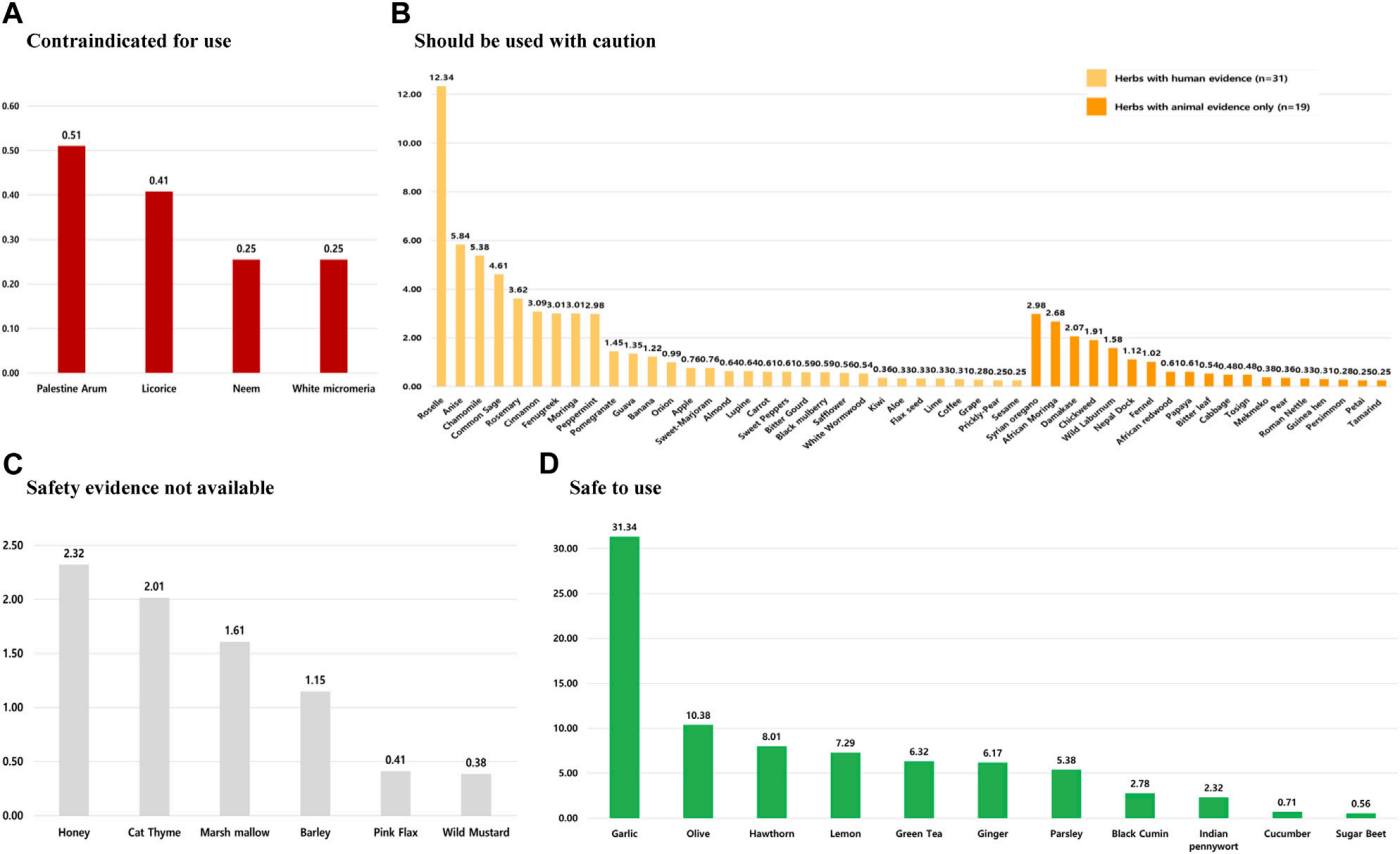
Prevalence of HMs used among hypertensive patients by safety classification. HMs in these sections are classified as follows: **(A)** can be potentially harmful to use, **(B)** should not be used without the supervision of a qualified healthcare practitioner, **(C)** safety evidence for herbal medicines not available, and **(D)** safe to use among hypertensive patients. *Note: The prevalence of each herb’s use was calculated by dividing the number of users for a particular herb by the total number of herbal medicine users.

Supplementary Table S5 provides a detailed analysis of the safety classification of commonly used HMs among patients with HTN. In this review, 71 herbs were identified, and four of them were classified as contraindicated based on four safety criteria. Notably, clinical evidence involving human subjects was available for two of these herbs (*Glycyrrhiza glabra* L. and *A. indica* A. Juss.), while the safety classification for *Arum palaestinum* Boiss. and *Micromeria fruticosa* (L.) Druce relied solely on animal studies. Furthermore, 50 herbs were categorized to be used with caution, while 11 herbs were classified as safe and suitable for HTN patients. Lastly, the safety of the remaining six herbs could not be determined due to insufficient evidence in the current literature for HTN patients (Figure 4).

## 4 Discussion

The present study presents the first systematic review and meta-analysis of HM use among HTN patients, identifying herbs commonly and globally used and assessing their safety based on current evidence. In total, 37 cross-sectional studies were included, with a combined sample size of 23,947 HTN patients. Of these, 37.8% reported using one or more types of HM during their treatment. The prevalence of HM utilization varied significantly by publication year and continent. Higher utilization rates were observed in studies published after 2011 (41.0%) than before 2011 (28.6%). Additionally, there were significant regional variations, with the highest use reported in North America (62.5%) and the lowest in Europe (15.7%). These regional differences may be attributed to various factors, including geographical characteristics, social and cultural influences, sociodemographic characteristics of study participants, and the quality of conventional therapy (Liwa et al., 2014; Peltzer and Pengpid, 2018; Azizah et al., 2021). Consequently, further research is warranted to explore the potential associations among these factors.

Patients with HTN commonly turn to HM to manage a range of physical conditions. The most frequently reported reasons for using HM include controlling blood pressure (BP), managing common comorbidities associated with HTN, and alleviating side effects from prescription drugs, such as frequent urination, headaches, and fatigue (Joshi et al., 2010; Olowofela and Isah, 2017). Additionally, some patients use HM to enhance their overall health status and wellbeing. The decision to use HM among HTN patients is often based on the belief that HM is safer and has fewer adverse effects than conventional antihypertensive drugs. Some patients opt for non-conventional treatment modalities due to concerns about the potential toxicity or dissatisfaction with conventional medicine in meeting their healthcare needs (Ahmed et al., 2017).

Despite the widespread use of HM among HTN patients, previous reviews on HM use among HTN patients have overlooked the evaluation of the safety of commonly used herbs. Thus, this study evaluated the safety profiles of the 71 most common HMs used by HTN patients, as identified in the included studies. The results revealed that four herbs were contraindicated for use, while 11 herbs were considered safe for use. Of the four herbs classified as contraindicated, two had no evidence of efficacy in lowering BP, and the other two herbs, *G. glabra* and *A. indica*, were supported only by a limited number of animal studies and had no clinical evidence of efficacy (Khoshnam and Bahaoddini, 2013; Shah et al., 2014). While these animal studies demonstrated potential benefits in controlling BP and managing common comorbidities associated with hypertension, numerous adverse effects were observed. For example, the use of *G. glabra* was associated with increased BP, hypokalemic-induced secondary disorders, rhabdomyolysis, acute renal failure, metabolic alkalosis, acute tubular necrosis, uremic, and paralysis in cases of chronic use (Van Uum, 2005; Sontia et al., 2008; Nazari et al., 2017; Penninkilampi et al., 2017; Kwon et al., 2020). Similarly, the misuse of *A. indica* was linked to severe stomatitis, marked oliguria, sanguineous vomiting, and even death (Wajdy et al., 2021). As a result, the consumption of these herbs or supplements containing their extracts should be avoided for HTN patients. These results stress the importance of safety in using HM for HTN management and highlight the need for evidence-based recommendations to enhance healthcare practices for HTN patients.

Amidst the potential risks, several herbs have been identified to provide clinical benefits while also being safe for use. *A. sativum*, used by 31.3% of HTN patients worldwide, is a popular HM used for managing HTN. Patients turn to *A. sativum* not only for HTN control but also for managing diabetes mellitus, alleviating prescription drug side effects, and improving overall health. Numerous studies and reviews have reported the antihypertensive effects of *A. sativum*, with a mean reduction in systolic/diastolic BP of 8.3/5.5 mmHg observed after administration of various *A. sativum* preparations and doses ranging from 600 mg/day to 1,200 mg/day over a median follow-up of 12 weeks (Shouk et al., 2014; Chrysant and Chrysant, 2017; Ried, 2020; EKİCİ et al., 2023). Its use has not been linked to any harmful events among HTN patients, although caution is advised with higher doses to prevent minor gastrointestinal disturbances (Matsutomo, 2020).

Similarly, *O. europaea* and *C. oxyacantha* are HMs that are commonly used by HTN patients and are known for their safety and efficacy in managing HTN at recommended doses, with no significant side effects reported (Susalit et al., 2011; Cloud et al., 2020; Venkatakrishnan et al., 2020; Ismail et al., 2021; Sun Y. et al., 2022; Johnson-Moore et al., 2023). The mechanism of action of *O. europaea* involves ACE inhibition, Ca^2+^ channel blockade, vasodilation, and antioxidant effects of flavonoids such as quercetin and rutin. Clinical trials administering 500 mg/day of *O. europaea* leaf extract *versus* placebo or no treatment resulted in a significant reduction in systolic/diastolic BP of 11.5/4.8 mmHg over 8 weeks (EKİCİ et al., 2023; Álvares et al., 2024). The beneficial effects of 900 mg/day *C. oxyacantha* on HTN have been consistently reported, where significant reductions in both SBP and DBP of approximately 17.2 mmHg and 9.2 mmHg, respectively, have been observed, especially when used for at least 12 weeks. These effects are primarily attributed to its flavonoids and oligomeric proanthocyanidins. Specifically, quercetin, the major polyphenolic flavonoid in *C. oxyacantha*, has shown efficacy in reducing BP through its antioxidant, anti-inflammatory, and vasorelaxant properties (Al-Gareeb, 2012; Cloud et al., 2020). Compounds found in *C. limon* (hesperidin and naringin) and *C. sinensis* (catechin) also act as vasodilators with antioxidant, anti-inflammatory, and antihypertensive properties, contributing to the use of HM in HTN patients (Peng et al., 2014; Rawat et al., 2016; David, 2017; Shilpa and Souza, 2020).

Fifty herbs have been identified for use with caution, as some of them have been associated with serious adverse effects, while others lack sufficient human evidence to determine their safety, particularly in HTN patients. *H. sabdariffa*, the second most commonly used HM among HTN patients, is frequently used to manage mild ailments and lower BP, and clinical literature reported no harmful effects in HTN patients (McKay et al., 2010; Serban et al., 2015). However, caution is still advised when using *H. sabdariffa*, as some studies reported diuretic effects and hepatotoxicity associated with high doses (Hopkins et al., 2013; Diallo et al., 2019). Also, *P. anisum* L. and *Salvia officinalis* L. are generally considered safe, but high doses or frequent intake of *P. anisum* seeds and its oil may cause nausea, vomiting, and pulmonary edema (Singletary, 2022), and excessive use of *S. officinalis* with high thujone content can lead to allergic reactions in some individuals (Mills and Bone, 2004; Hamidpour et al., 2014). In addition, although the consumption of *Stellaria media* L. tea and *Foeniculum vulgare* Mill. has not shown any toxicity or adverse events in animal studies, their safety and efficacy in HTN patients have yet to be investigated. As a result, it is recommended to use these herbs under the supervision of a qualified healthcare practitioner.

Finally, the safety of six HMs, such as honey, *Teucrium polium* L., and *Althaea officinalis* L., could not be established due to the lack of scientific research on their potential toxicity in humans (Reinelt and Melzig, 2017; Kianitalaei et al., 2019; Akhbari et al., 2021; Gholami et al., 2022). Therefore, it is recommended to avoid using these HMs until clinical evidence is available to ensure safety. In addition, certain substances that are commonly used in foods, such as honey, *A. officinalis*, and *Hordeum vulgare* L., should only be consumed in moderation and in amounts typical of culinary practices because excessive consumption of these HM may result in potential adverse effects. These findings emphasize the importance of conducting clinical studies to establish the toxicity and safety of commonly used herbs.

Before interpreting the findings of this systematic review, it is important to consider the following limitations. First, the reviewed studies were selected from three databases and restricted to articles published in the English language, which many potentially limit the generalizability of findings regarding the utilization of HM among HTN patients. Second, significant variations in sample size and study settings were observed among the reviewed studies, with the sample size ranging from 74 to 4,575 participants and the study settings varying from primary healthcare centers to tertiary teaching hospitals. The number of hospitals surveyed varied from a single institution to as many as 16 facilities (Mahfudz and Chan, 2005; Clement et al., 2007; Ali-Shtayeh et al., 2013; Boima et al., 2015).

The extent of HM use investigated also varied among the studies, with four studies excluded from the final analysis as they only investigated whether patients used HM or not and did not report the number of users for individual HM modalities (Mahfudz and Chan, 2005; Wazaify et al., 2013; Boima et al., 2015; Owusu et al., 2020). Lastly, due to variations in study quality, numerous studies were omitted from the final analysis during the risk of bias assessment phase. Initially, 48 studies from 25 countries were considered for the review, but after the quality assessment of the studies, 37 cross-sectional studies from 21 countries remained for the final analysis. Hence, it is essential to consider the discrepancies when interpreting the results of this review, and additional studies with a focus on methodological rigor are needed to achieve a more comprehensive understanding of HM use among HTN patients globally. Despite these limitations, our findings offer valuable insights into the safety profile of HMs commonly used by HTN patients, facilitating the development of evidence-based guidance for policymakers and healthcare providers involved in HTN management.

## 5 Conclusion

The use of HM among HTN patients is widespread globally. Our study emphasizes the significance of recognizing the risks of toxicities and adverse effects associated with HM use for HTN treatment. To prioritize patient safety, it is crucial to take only HM classified as safe in normal doses and avoid contraindicated HM. The safety classification in this review can raise awareness among physicians and healthcare providers regarding potential adverse effects in HTN patients. Future research should prioritize identifying commonly used herbs among HTN patients, particularly in resource-limited countries with poor HTN management. Conducting additional clinical studies is imperative to thoroughly evaluate the toxicity and safety of these medicinal plants.

## Data Availability

All data generated and analyzed, including study protocol, search strategy, list of included and excluded studies, extracted data, analysis plans, quality assessment, and assessment of publication bias, will be made available by the authors upon reasonable request. Requests to access these datasets should be directed to the corresponding author.

## Abbreviations

BP, blood pressure; CAM, complementary and alternative medicine; CI, confidence interval; DM, diabetes mellitus; HM, herbal medicine; HTN, hypertension; RCT, randomized controlled trial.
