# Supplementary figures and images for: Safety classification of herbal medicine use among hypertensive patients: a systematic review and meta-analysis
Source: Front Pharmacol. 2024 May 31;15:1321523. doi: 10.3389/fphar.2024.1321523 (PMC11176523; doi:10.3389/fphar.2024.1321523)

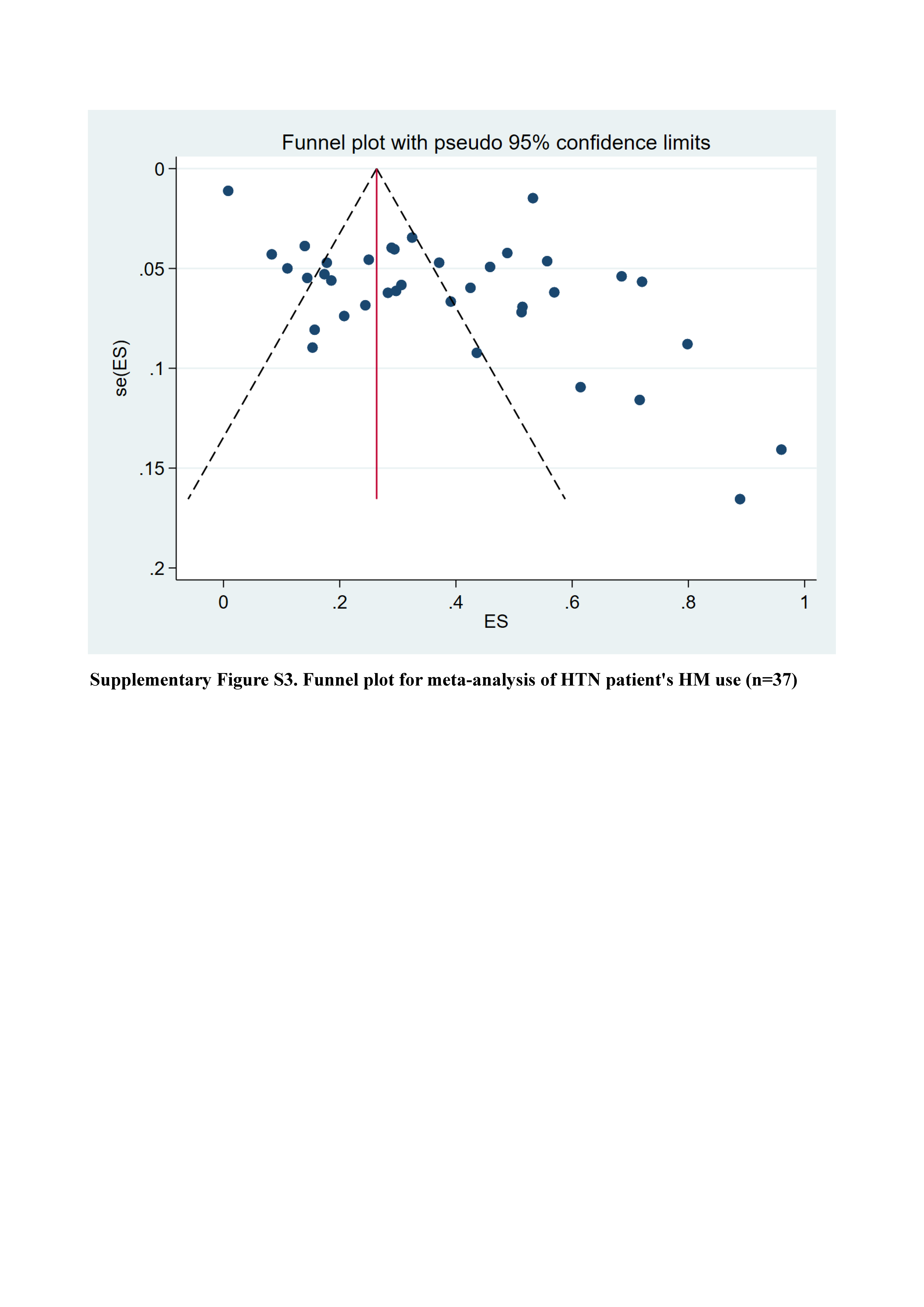

Supplement: Supplementary file 2 [file Image1.tif]
