# Supplementary material for: Safety classification of herbal medicine use among hypertensive patients: a systematic review and meta-analysis
Source: Front Pharmacol. 2024 May 31;15:1321523. doi: 10.3389/fphar.2024.1321523 (PMC11176523; doi:10.3389/fphar.2024.1321523)
Supplement: Supplementary file 3 [file Table2.docx]

Supplementary Table S2. Search strategy

| Categories | Contents | | |
| --- | --- | --- | --- |
| Database | PubMed/MEDLINE | EMBASE (Ovid) | CINAHL |
| Search date | 19 June 2023 | | |
| Date | Database inception to 2023 | | |
| Search terms | **S1** ((hypertension[MeSH Terms]) OR (Herbal medicine[MeSH Terms]) OR (phytotherapy[MeSH Terms]) OR (cross sectional studies[MeSH Terms])) **AND**  **S2** (High Blood Pressure) OR (hypertensi*) **AND**  **S3** ((herbal preparations) OR (herbal tea) OR (herbal remed*) OR (herbal therap*) OR (herbal product*) OR (medicinal herb) OR (medicinal plant) OR (phytomedicine)) **AND**  **S4** ((cross sectional study) OR (cross sectional survey) OR (survey)) | **S1** ((herbal medicine/ OR herbal tea/ OR herb/ OR medicinal plant/ OR phytotherapy/ OR herbal remed*.mp. OR herbal therap*.mp. OR herbal product*.mp.)  **AND**  **S2** (exp hypertension/ OR hypertensi*.mp.)  **AND**  **S3** (cross-sectional study/ OR cross sectional survey.mp. OR survey.mp.)) | **S1** ((herbal medicine OR herbal tea OR herb OR medicinal plant OR phytotherapy OR herbal remed* OR herbal therap* OR herbal product*)  **AND**  **S2** (hypertension/ OR hypertensi*)  **AND**  **S3** (cross-sectional study OR cross sectional survey OR survey)) |
