# Supplementary material for: Safety classification of herbal medicine use among hypertensive patients: a systematic review and meta-analysis
Source: Front Pharmacol. 2024 May 31;15:1321523. doi: 10.3389/fphar.2024.1321523 (PMC11176523; doi:10.3389/fphar.2024.1321523)
Supplement: Supplementary file 4 [file Table5.docx]

Supplementary Table S5. Safety classification of commonly used herbal medicines among HTN patients

| No. | Herbal medicines (n=71) | Effectiveness^a^ | Study subjects^b^ | | Documentation on safety |
| --- | --- | --- | --- | --- | --- |
|  |  |  | **Animal** | **Human** |  |
| Contraindicated for use (4) | | | | | |
| 1 | *Arum palaestinum* Boiss. | ᅳ | ✕ | ᅳ | Despite showing anti-cancer effects, there is no clinical evidence for its safety or adverse effects in HTN patients (Hatmal et al., 2017; Kmail et al., 2022). An animal study suggests potential liver toxicity (Al-Qudah, 2016), thus contraindicating it for HTN patients. |
| 2 | *Glycyrrhiza glabra* L. | ○ | ✕ | ✕ | While there is currently no clinical evidence available, an animal study demonstrated that *G. glabra* exhibits a hypotensive effect on BP by modulating the adrenergic system and synergizing with the cholinergic system (Khoshnam and Bahaoddini, 2013). However, in humans, one of the major side effects of *G. glabra* is HTN and hypokalemic-induced secondary disorders. Chronic use of *G. glabra* can cause rhabdomyolysis, acute renal failure, metabolic alkalosis, acute tubular necrosis, uremic, and even paralysis (Van Uum, 2005; Sontia et al., 2008; Nazari et al., 2017; Penninkilampi et al., 2017; Kwon et al., 2020). Therefore, HTN patients should avoid the consumption of *G. glabra* or supplements containing *G. glabra* extract. |
| 3 | *Azadirachta indica* A. Juss. | ○ | ✕ | ✕ | Animal studies have indicated that the vasodilator and cardiac depressant properties of *A. indica* may contribute to lowering BP when consumed (Shah et al., 2014). Nevertheless, incidences of severe stomatitis, marked oliguria, sanguineous vomiting, and even death have occurred in humans when the bark and leaves of *A. indica* are misused (Wajdy et al., 2021). Hemolytic anemia with jaundice and dizziness after high-dose of herbal intake (tea) has also been observed in patients with T2DM (Islas et al., 2020). |
| 4 | *Micromeria fruticosa* (L.) Druce | ᅳ | ✕ | ᅳ | Despite showing antitumor activities, no scientific reports currently exist regarding the utilization and safety of *M. fruticosa* in HTN patients. An animal study has indicated a high level of toxicity, with a 50% mortality rate observed at a dose of 52 mg/kg (Shehab and Abu-Gharbieh, 2012) This level of toxicity strongly advises against the use of *M. fruticosa* in high doses for individuals with HTN. |
| Should be used with caution (50) | | | | | |
| 1 | *Hibiscus sabdariffa* L. | ○ | ᅳ | △ | Clinical trials have demonstrated that treatment with dried extract of *H. sabdariffa* ranging from 240 ml to 10 g for up to 6 weeks leads to significant reductions in SBP and DBP, attributed to increased nitric oxide production, calcium channel blocking, and opening of ATP-sensitive K+ channels (Kamyab et al., 2021). Also, in the current clinical literature, no harmful effects of *H. sabdariffa* were found on HTN patients (McKay et al., 2010; Serban et al., 2015), yet an adverse diuretic effect was found in another study (Diallo et al., 2019). Furthermore, hepatotoxicity was observed in extremely high doses (Hopkins et al., 2013). |
| 2 | *Pimpinella anisum* L. | ○ | ᅳ | △ | *P. anisum* and *P. anisum* oil are generally safe, but high doses or intake frequency may cause adverse effects like nausea, vomiting, and pulmonary edema. As the impact on HTN patients is not well-understood, it's recommended to consume it at typical food levels (Singletary, 2022). |
| 3 | *Matricaria chamomilla* L. | ○ | ○ | △ | *M. chamomilla* extract has been shown to lower BP in both rats and humans with HTN. However, there is limited evidence regarding its safety in patients with HTN (Shoara et al., 2015; Awaad et al., 2018; da Silva et al., 2022; Dai et al., 2022; Sah et al., 2022). Some studies have reported allergic reactions, such as contact dermatitis and hypersensitivity, in individuals allergic to pollen or composite (Dai et al., 2022). Therefore, caution is advised when using this herb. |
| 4 | *Salvia officinalis* L. | ○ | ᅳ | △ | Human studies have indicated that normal usage of *S. officinalis* is safe. However, there might be an adverse effect (i.e., allergic reaction) on using *S. officinalis* in excessive amounts due to the high thujone content (Mills and Bone, 2004; Hamidpour et al., 2014). Therefore, it should only be used with the proper supervision of a qualified healthcare practitioner. |
| 5 | *Salvia rosmarinus* L. | ○ | ᅳ | △ | The volatile *S. rosmarinus* oil increased BP in patients diagnosed with primary hypotension. Therefore, *S. rosmarinus* products should be used cautiously in patients with HTN and DM (Hassani et al., 2016). |
| 6 | *Cinnamomum verum* J. Presl. | ○ | ᅳ | △ | Both animal and clinical studies have shown the BP lowering effects of *C. verum* by increasing nitric oxide levels (Kamyab et al., 2021). A systematic review found that *C. verum* intake within the daily range did not have significant toxic or adverse effects in clinical trials. Among DM patients, no clinically severe adverse events were observed except for minor gastrointestinal events and skin rash cases among DM patients (Gu et al., 2022; Zhou et al., 2022). Therefore, its use is acceptable in a moderate amount, but due to limited evidence on HTN patients, it should be used with caution. |
| 7 | *Trigonella foenum-graecum* L. | ○ | ᅳ | △ | There is a lack of evidence regarding *T. foenum-graecum* seed consumption in HTN patients, yet, no notable side effects have been reported from consuming *T. foenum-graecum* seed in DM patients (Ulbricht et al., 2008; Bahmani et al., 2016). |
| 8 | *Moringa oleifera* L. | ○ | ᅳ | △ | Oral treatment with *M. oleifera* leaf extract (30 and 60 mg/kg/day) has been shown to reduce both SBP and DBP in a dose-dependent manner by alleviating vascular dysfunction and oxidative stress, while also promoting endothelium-dependent vasorelaxation (Aekthammarat et al., 2019; Kamyab et al., 2021). Regardless of its form (i.e., powder or aqueous extract), human and animal toxicity studies showed that *M. oleifera* is safe, with no adverse effects reported (Stohs and Hartman, 2015; Popoola et al., 2020). Thus, it can be used in moderate amounts, yet due to the limited evidence on HTN patients; it should be used with caution. |
| 9 | *Mentha piperita* L. | ○ | ᅳ | △ | No severe toxicity and adverse effects were reported from *M. piperita* use except for minor cases of heartburn and irritable bowel syndrome in a few human studies. However, *M. piperita* oil is contraindicated in patients with severe liver damage. It should also be used with caution in patients with gastrointestinal reflux or hiatus hernia because it may exacerbate the symptoms of gastrointestinal reflux (Mahendran and Rahman, 2020; Malekmohammad et al., 2021). |
| 10 | *Majorana syriaca* (L.) Rafin. | ᅳ | △ | ᅳ | While the antihypertensive effects of *M. syriaca* remain unexplored, its antioxidant properties could indirectly contribute to lowering BP and preventing cardiovascular complications (Husein et al., 2014). Although toxicity studies in animals have demonstrated the safety of consuming this plant, it's essential to exercise caution when using this herb due to the absence of human evidence (Mesmar et al., 2022). |
| 11 | *Punica granatum* L. | ○ | ᅳ | △ | In clinical studies, consumption of *P. granatum* juice (50-150 ml/d) reduced SBP by 5-7%, DBP by 6%, and serum ACE activity by 36%, and it may offer protection against cardiovascular diseases by inhibiting serum ACE activity and reducing oxidative stress (EKİCİ et al., 2023). Existing evidence shows no adverse effects from its use in humans (Laurindo et al., 2022). However, although no harmful events have been identified in its use and safety in the current literature, additional clinical trials are needed to determine the correct formulations and doses to prevent potential adverse effects (Hosseini et al., 2022). |
| 12 | *Psidium guajava* L. | ○ | ᅳ | △ | Toxicity reviews, including both animals and humans, on several parts and extracts of *P. guajava* showed that it is safe for consumption without any side effects (Kamath et al., 2008). However, it should be used with caution as some studies reported potential toxic effects when excessive phytochemicals are ingested (Takeda et al., 2022). |
| 13 | *Musa paradisiaca* L. | ○ | ○ | △ | Toxicity studies in animal models and human studies show that the leaves and fruits of *M. paradisiaca* are safe for use without side effects (Ugbogu et al., 2018; Costa et al., 2019; Ajijolakewu et al., 2021; Mondal et al., 2021). Nevertheless, further studies at the clinical level are required to establish the clinical efficacy and safety of *M. paradisiaca* use in HTN patients. |
| 14 | *Allium cepa* L. | ○ | △ | △ | *A. cepa* has demonstrated BP-lowering effects in animal studies, attributed to its organo-sulfur compounds, which maintain arterial elasticity and reduce blood viscosity, thereby preventing blood clotting. Furthermore, quercetin in *A. cepa* can lower BP by approximately 5 mmHg through its antioxidant properties and promotion of vascular function (Kamyab et al., 2021). Although no potential side effects of *A. cepa* were identified in human review studies, an animal study found that *A. cepa* consumption could result in hemolytic anemia (Chakraborty et al., 2022); thus, it should not be used in doses higher than commonly found in food and be used with caution (Zhao et al., 2021). |
| 15 | *Origanum majorana* L. | ○ | ○ | △ | The toxicological investigation of *O. majorana* confirmed its safety, as several reviews of animal studies found no toxicity or potential side effects (Tripathy et al., 2017; Bouyahya et al., 2021). Also, human evidence shows that it can be safely consumed as a tea in moderate amounts (Haj‐Husein et al., 2016), but no evidence is available regarding HTN patients to date; thus, it should be used with caution. |
| 16 | *Malus domestica* Borkh. | ○ | △ | △ | The safety of *M. domestica* consumption has only been studied on DM patients, and no direct evidence is available for HTN patients. However, its use is considered generally safe and non-fatal, and only mild side effects such as headache and rash are common (Kim et al., 2022; Patil et al., 2022). |
| 17 | *Prunus dulcis* L. | ○ | ᅳ | △ | Reviews of human studies demonstrated no serious adverse effects or toxic reactions from *P. dulcis* ingestion (Karimi et al., 2021). However, high consumption of *P. dulcis* can cause side effects such as allergies, nausea, and digestive problems (Luo et al., 2023). In addition, the high oxalate content in nuts, such as *P. dulcis*, could be a risk factor contributing to kidney stone formation in some individuals (Eslampour et al., 2020). Therefore, its use should be limited to an acceptable amount and be used with caution. |
| 18 | *Lupinus albus* L. | ○ | ᅳ | △ | A review study showed that *L. albus* is safe to use in humans (Ishaq et al., 2022), yet no direct evidence is available regarding its use among HTN patients. Therefore, it should be used with caution. |
| 19 | *Capsicum annuum* L. | ○ | △ | △ | All RCTs on *C. annuum* intake reported no serious adverse effects, but some intervention groups experienced adverse effects such as leg cramps, skin irritation, neurotoxicity, dyspepsia, bowel irregularities, diarrhea, and skin rash. Thus, caution is advised when using them (Srinivasan, 2016; Sanati et al., 2018; Jang et al., 2020; Shirani et al., 2021). |
| 20 | *Daucus carota* L. | ○ | ᅳ | △ | *D. carota* is effective in controlling BP due to its potassium content and antioxidant-rich nature, which reduces oxidative stress and supports blood vessel function and structure (Kamyab et al., 2021). Despite its antihypertensive properties, it should be used cautiously in patients with hypoglycemia, diabetes, hormone-sensitive conditions, or bowel obstruction. Additionally, *D. carota* intake can increase gastrointestinal transit time, so patients taking medication orally must be careful (Ulbricht, 2010; Akhtar et al., 2017; Kaur, 2019). |
| 21 | *Morus nigra* L. | ○ | △ | **△** | *M. nigra* is safe for diabetic patients and animals, despite no safety evidence for HTN patients (Lim and Choi, 2019; Park et al., 2019; Zhang et al., 2022; Cui et al., 2023). Moreover, systematic reviews and RCTs found no adverse events or toxic effects except mild gastrointestinal symptoms (Thaipitakwong et al., 2020). |
| 22 | *Momordica charantia* L. | ○ | ᅳ | △ | Human studies have indicated that the consumption of *M. charantia* could result in minor side effects, such as fatigue, dizziness, and pruritus (Kim et al., 2023a). Among T2DM patients, no clinically severe adverse events were observed, yet minor gastrointestinal problems (i.e., abdominal pain, diarrhea, nausea, and soreness) occurred (Kim et al., 2020). However, due to the lack of evidence on HTN patients, it should not be used without the proper supervision of a qualified healthcare professional (Basch et al., 2003). |
| 23 | *Carthamus tinctorius* L. | ○ | △ | △ | *C. tinctorius* has been shown to lower BP by opening ATP-sensitive K+ channels and reducing renin activity and angiotensin II levels in rats (Kamyab et al., 2021). Although *C. tinctorius* use has been shown to also improve BP in patients with metabolic syndrome (Ruyvaran et al., 2022), further research is needed to evaluate its safety in HTN patients, potential interactions with anti-hypertensive drugs, and identify any side effects or toxicity (Zhou et al., 2014; Bunbupha et al., 2019; Yu et al., 2019; Orgah et al., 2020). |
| 24 | *Artemisia herba-alba* Asso. | ○ | ᅳ | △ | *A. herba-alba* improves animal BP, but caution is required in humans due to potential side effects such as ulcers or allergies at high doses (Al-Waili, 1986; Zeggwagh et al., 2008). Thus, it should be used under the supervision of a qualified healthcare practitioner (Benkhaira et al., 2021). |
| 25 | *Actinidia deliciosa* A. Chev | ○ | ○ | △ | HTN patients have shown antihypertensive effects, with decreased BP and ACE inhibition (Mohajeri et al., 2014; Svendsen et al., 2015; Tyagi et al., 2015; Zhao et al., 2017; Prasad et al., 2020), and no reported side effects in human studies (Duttaroy and Jørgensen, 2004; Richardson et al., 2018). However, due to the lack of safety evidence for HTN patients, caution should be exercised when using it. |
| 26 | *Citrus aurantiifolia* (Christm.) Swingle | ○ | △ | △ | An animal study on *C. aurantiifolia* found that its methanol extract has antihypertensive effects (Enejoh et al., 2015; Rana et al., 2022). However, it's worth noting that *C. aurantiifolia* essential oil, considered safe, can have mild hematotoxic, nephrotoxic, and hepatotoxic effects in rats (Dosoky and Setzer, 2018; Adokoh et al., 2019). While generally safe for humans with negligible toxicity, there is currently no safety evidence for HTN patients, so its use should be supervised by a qualified healthcare practitioner. |
| 27 | *Linum usitatissimum* L. | ○ | ᅳ | △ | *L. usitatissimum* was found effective in reducing BP, cholesterol, and body mass index in HTN patients (Ursoniu et al., 2016; Toulabi et al., 2022). However, prolonged or excessive consumption can cause severe neurological symptoms like paralysis or seizures. Inadequate water intake with *L. usitatissimum* can result in intestinal obstruction. *L. usitatissimum* oil consumption may lead to adverse effects such as generalized urticaria, ocular pruritus/weeping, nausea/vomiting, and intestinal/abdominal pain. The consumption of immature *L. usitatissimum* seedpods should be avoided as they may be poisonous (Basch et al., 2007; Ulbricht, 2010). |
| 28 | *Aloe vera* (L.) Burm.f. | ○ | ○ | △ | Several clinical trials evaluated the efficacy of *A. vera* supplementation in various diseases. There is no direct evidence on HTN patients, but it was indicated safe for diabetic patients and other patient groups with no significant side effects (Radha and Laxmipriya, 2015; Sabbaghzadegan et al., 2021; Yaqoob et al., 2021). |
| 29 | *Coffea arabica* L. | ○ | ○ | △ | Moderate *C. arabica* intake (1-3 cups/day) is generally safe and may have benefits in managing HTN, including a reduced risk of all-cause and cardiovascular mortality in elderly HTN patients (Hutachok et al., 2021; Surma and Oparil, 2021; Chen et al., 2022; Yamaji et al., 2022). However, some studies on HTN patients have yielded inconsistent results. Therefore, to avoid adverse effects, HTN patients with uncontrolled BP should limit their consumption of high doses of caffeine (Palatini et al., 2016; Rodríguez-Artalejo and López-García, 2017; Shah et al., 2023). |
| 30 | *Vitis vinifera* L. | ○ | ○ | △ | Both clinical trials and animal studies have confirmed the beneficial effects of *V. vinifera* polyphenols in lowering BP and its antihypertensive properties (Akaberi and Hosseinzadeh, 2016; Sochorova et al., 2020; Foshati et al., 2022; Majeed et al., 2022). No severe toxicity or adverse effects have been reported from *V. vinifera* use, but a human study indicated that adverse gastrointestinal effects appeared with doses of 2.5 and 5 g (Nassiri‐Asl and Hosseinzadeh, 2009; 2016). Therefore, toxicological studies are needed for *V. vinifera* product doses, and more clinical trials are required. |
| 31 | *Opuntia ficus-indica* (L.) Mill. | ○ | ○ | △ | Research on rats and rabbits has revealed that the extract of *O. ficus-indica* exhibits diuretic and hypotensive effects (Bakour et al., 2017; Han et al., 2019). To date, there have been no reports of adverse or toxic effects on humans (Osuna-Martínez et al., 2014; Onakpoya et al., 2015). Nevertheless, some studies have noted mild adverse effects like diarrhea, gastric intolerance, and flu-like symptoms (Osuna-Martínez et al., 2014; Onakpoya et al., 2015; Martins et al., 2023). There is currently no direct evidence regarding its effects on HTN patients. Consequently, additional clinical trials are necessary to evaluate its impact on body composition and cardiovascular profile. |
| 32 | *Sesamum indicum* L. | ○ | ○ | △ | Both animal models and human studies have demonstrated the anti-hypertensive effects of *S. indicum*, and no toxicity has been reported with *S. indicum* seed oil (Cardoso et al., 2018; Gebrekidan and Desta, 2019; Huang et al., 2022; Wei et al., 2022). However, there is limited evidence regarding its safety in HTN patients. Therefore, caution should be exercised when using *S. indicum* in individuals with gastrointestinal disorders (Rouhi-Boroujeni et al., 2017). |
| 33 | *Moringa stenoptela* (Baker f.) Cufod | ○ | ○ | ᅳ | Animal evidence suggests *M. stenoptela* has anti-hypertensive effects, but increased doses showed mild toxicity (Mengistu et al., 2012; Geleta et al., 2016; Hadis et al., 2020). As human evidence is lacking, further safety evaluation is necessary before considering its use (Seifu, 2015). |
| 34 | *Ocimum lamiifolium* Hochst. ex Benth. | ᅳ | ○ | ᅳ | While *O. lamiifolium* did not show a direct effect on lowering BP, an animal study revealed its anti-inflammatory properties (Mequanint et al., 2011). When administered to healthy rats, this herb demonstrated no significant adverse effects at normal doses. However, caution is necessary as high doses were found to be hepatotoxic and carcinogenic. Thus, the use of high doses of *O. lamiifolium* should be avoided (Adane et al., 2023). |
| 35 | *Stellaria media* L. | ᅳ | ○ | ᅳ | *S. media* tea may protect against diabetes-induced cardiac dysfunction and is considered safe and non-toxic in animal studies. However, its use and safety among HTN patients are yet to be investigated; therefore, it should be only used with the supervision of a qualified health care practitioner (Demján et al., 2022; Singh et al., 2022). |
| 36 | *Calpurnia aurea* (Ait.) Benth. | ○ | ○ | ᅳ | An animal study demonstrated that *C. aurea* effectively lowers BP by dilating blood vessels without any significant toxic effects. However, to ensure its safe clinical application, further evaluation of its safety on HTN patients is necessary (Umer et al., 2013; Birhanu et al., 2015; Getiye et al., 2016; Ayal et al., 2019; Andargie et al., 2022). |
| 37 | *Rumex nepalensis* Spreng. | ᅳ | ○ | ᅳ | No specific investigation into the direct BP lowering effect and safety of *R. nepalensis* has been conducted in humans. Animal studies have shown conflicting findings regarding its toxicity and safety (Belsty et al., 2019; Gonfa et al., 2021). Further investigation is necessary to establish its safety for patients with HTN. |
| 38 | *Foeniculum vulgare* Mill. | ○ | ○ | ᅳ | No scientific report was available on its use and safety among HTN patients. In several animal studies, no severe toxicity or adverse events occurred after using *F. vulgare* except for minor allergic reactions (Bardai et al., 2001; Rahimi and Ardekani, 2013; Hong et al., 2022). |
| 39 | *Hagenia abyssinica* J.F. Gmel. | ᅳ | ○ | ᅳ | *H. abyssinica* has antidiabetic effects, but its antihypertensive effects are unexplored. Animal studies on diabetic rats show no visible toxicity (Kifle and Belayneh, 2020; Kifle et al., 2020), while an ethnomedical study reported adverse effects, including gastrointestinal upset, abortion, and death (Assefa et al., 2010). More research is needed to understand its safety and efficacy for HTN. |
| 40 | *Carica papaya* L. | ○ | △ | ᅳ | *C. papaya* is effective for managing HTN (Eno et al., 2000; Brasil et al., 2014; Wahdi et al., 2020; HASIMUN, 2022), but its safety is not fully established due to conflicting findings from animal studies showing hepatotoxicity (Eno et al., 2000; Santana et al., 2019). Prolonged consumption of this fruit may cause infertility, and its leaves and roots contain potentially fatal cyanogenetic glucosides (Gunde and Amnerkar, 2016). Therefore, its use should be supervised by healthcare professionals to ensure safety. |
| 41 | *Vernonia amygdalina* Delile | ○ | ○ | ᅳ | Although animal toxicity studies suggest *V. amygdalina* is safe in moderate quantities (Taiwo et al., 2010; Olaiya et al., 2013; Onyema-iloh et al., 2018; Putri et al., 2022), its safety in HTN patients has not been established. Caution is advised when using *V. amygdalina*. |
| 42 | *Brassica oleracea* L. | ○ | ○ | ᅳ | Although animal studies showed no toxicity in ethanolic extract of *B. oleracea* at a dose of ≥ 2,000 mg/kg, its safety in HTN patients remains unknown. Therefore, caution is advised when using *B. oleracea* (Assad et al., 2014; Uuh‐Narvaez and Segura‐Campos, 2021; Asiwe et al., 2022). |
| 43 | *Thymus schimperi* R. | ᅳ | ○ | ᅳ | *T. schimperi* leaves have been found to lower blood glucose levels in diabetic mice, but their antihypertensive effects remain unexplored. Safety-wise, extracts showed no acute toxicity at a 2 g/kg dose (Damtie et al., 2017; Melesie Taye et al., 2020). However, an *in silico* study revealed potential hepatotoxicity and mitochondrial membrane toxicity of a few components in the oil (Adane et al., 2021). Therefore, consuming its plant parts in regular food amounts is considered non-toxic, yet using its oil in high doses should be avoided, and supervised use is recommended. |
| 44 | *Rumex abyssinicus* Jacq. | ᅳ | ○ | ᅳ | An animal study found that orally taken extracts of *R. abyssinicus* were safe and non-toxic (Mekonnen et al., 2010). However, its effects on HTN patients remain unknown. |
| 45 | *Pyrus communis* L. | ○ | ○ | ᅳ | Human studies have shown that *P. communis* effectively reduces BP, but there is a lack of safety evidence on human subjects. However, a study on diabetic rats found it to be safe up to doses of 2,000mg/kg (Velmurugan and Bhargava, 2013; Johnson et al., 2016). Additionally, *P. communis* contains a high level of fructose and sorbitol, which can be beneficial for most people, but it may cause issues for some individuals (James-Martin et al., 2015; Gayer et al., 2019; Navaei et al., 2019). |
| 46 | *Urtica pilulifera* L. | ᅳ | ○ | ᅳ | In the study on diabetic rats, *U. pilulifera* extracts showed no toxicity at doses up to 2 g/kg, and another animal study indicated showed no visible or histological side effects, indicating low toxicity and a very low risk for adverse events (Abo-elmatty et al., 2013; Aliwaini and Lubbad, 2016). However, further clinical studies are required to establish its safety for HTN patients. |
| 47 | *Petiveria alliacea* L. | ᅳ | △ | ᅳ | No scientific reports are available regarding the use and safety of *P. alliacea* in HTN patients. Conflicting findings from animal studies on its toxicity and safety necessitate further investigation to establish its safety (da PaixÃ et al., 2016; Luz et al., 2016; García-Pérez et al., 2018). Therefore, it should only be used under the supervision of a qualified healthcare practitioner. |
| 48 | *Diospyros kaki* L. | ○ | ○ | ᅳ | Animal study shows the hypotensive effects of unripened *D. kaki* (Liu et al., 2012; Xie et al., 2015). Animal studies on the leaves and fruit of *D. kaki* have not shown any side effects or toxicity reports (Xie et al., 2015; Ayşe and Ertuğrul, 2020). No scientific report was available on its use and safety among HTN patients. Therefore, further analysis is needed, focusing on different parts of the plant, such as the stem and fruit. |
| 49 | *Tamarindus indica* L. | ○ | ○ | ᅳ | Although the effectiveness of *T. indica* in reducing BP has been reported, its safety in HTN patients has not been studied (Toungos, 2019). However, animal studies have not reported any side effects from consuming *T. indica* (Ulbricht, 2010; Kuru, 2014; Meher et al., 2014; Komakech et al., 2019; Krishna et al., 2020). |
| 50 | *Parkia speciosa* Hassk. | ○ | ○ | ᅳ | Animal studies have indicated that *P. speciosa* is safe and non-toxic (Al Batran et al., 2013; Ahmad et al., 2019; Azemi et al., 2022). However, there is limited evidence regarding its safety in HTN patients. |
| Safety evidence not available (6) | | | | | |
| 1 | Honey | ○ | ─ | ─ | Both clinical and animal studies have demonstrated that administering honey significantly reduces both systolic and diastolic BP, likely due to its ability to alleviate oxidative stress and lower elevated BP. Despite its antihypertensive effects (Erejuwa et al., 2011; Aluko et al., 2013; Akhbari et al., 2021; Gholami et al., 2022), there is a lack of scientific reports on its safety for HTN. Further clinical studies are necessary to establish its toxicity and safety. |
| 2 | *Teucrium polium* L. | ○ | ─ | ─ | Although several animal studies have shown hypotensive effects of T. polium extract due to its calcium antagonist effect (Niazmand et al., 2011), no scientific report was available on its use and safety in HTN patients. |
| 3 | *Althaea officinalis* L. | ○ | ─ | ─ | An animal study demonstrated that administering *A. officinalis* extract effectively reduced BP by inhibiting the angiotensin-converting enzyme (Ziaei et al., 2009; Kianitalaei et al., 2019). Yet, limited literature is available to determine the toxicity or safety of *A. officinalis* in humans, so its safety among HTN patients cannot be determined (Reinelt and Melzig, 2017; Kianitalaei et al., 2019). |
| 4 | *Hordeum vulgare* L. | ○ | ─ | ─ | Animal studies have revealed that consumption of *H. vulgare* exerted anti-hypertensive effects attributed to its anti-oxidant, anti-inflammatory, and vasodilating properties (Sinha et al., 2012; Valenzuela et al., 2018; Ahmed-Farid et al., 2023), However, its safety remains inconclusive as it has not been studied in humans. |
| 5 | *Linum pubescens* Willd. ex Schult. | ─ | ─ | ─ | Although there is no direct clinical research on HTN patients, an ethnobotanical survey of folk toxic plants reported *L. pubescens* as a toxic plant (Al-Qura'n, 2005). |
| 6 | *Sinapis arvensis* L. | ─ | ─ | ─ | No scientific report was available on its use and safety among HTN patients. |
| Safe to use (11) | | | | | |
| 1 | *Allium sativum* L. | ○ | ─ | ○ | The BP-lowering effects of *A. sativum* have been reported in several studies and reviews; after a median follow-up of 12 weeks and using different *A. sativum* preparations and doses ranging from 600 to 1,200 mg/day, BP was lowered by a mean SBP/DBP of 8.3/5.5 mmHg (Shouk et al., 2014; Chrysant and Chrysant, 2017; Ried, 2020; EKİCİ et al., 2023). Thus, *A. sativum* is a safe and effective method for managing HTN in patients (Xiong et al., 2015b; Al Disi et al., 2016; Varshney and Budoff, 2016; Lucius, 2022), with only minor gastrointestinal disturbances reported from raw *A. sativum* consumption (Matsutomo, 2020). |
| 2 | *Olea europaea* L. | ○ | ─ | ○ | Administration of 500 mg per day of *O. europaea* leaf extract resulted in a significant reduction in SBP/DBP of 11.5/4.8 mmHg over 8 weeks compared with the placebo or no treatment groups (EKİCİ et al., 2023; Álvares et al., 2024). Review studies also show that moderate doses of oral *O. europaea* consumption is safe for HTN patients (Susalit et al., 2011; Ismail et al., 2021). |
| 3 | *Crataegus oxyacantha* L. | ○ | ○ | ○ | Clinical trials and systematic reviews have consistently reported the beneficial effects of *C. oxyacantha* in lowering BP through its antioxidant, anti-inflammatory, and vasorelaxant properties, resulting in significant reductions in both SBP and DBP, averaging approximately 17.2 and 9.2 mmHg, respectively, especially when used for at least 12 weeks (Al-Gareeb, 2012; Cloud et al., 2020). *C. oxyacantha* has been shown to be safe and effective for HTN at therapeutic doses, with no significant side effects reported (Cloud et al., 2020; Venkatakrishnan et al., 2020; Sun et al., 2022a; Johnson-Moore et al., 2023). However, it is not recommended as a standalone treatment for heart failure based on a review study (Djordjević and Nikolić, 2021). |
| 4 | *Citrus limon* L. | ○ | ─ | ○ | Consuming 10 ml of citric acid from *C. limon* resulted in a significant decrease in both SBP and DBP by an average of 13.4/9.6 mmHg after 30 days compared to baseline levels (David, 2017; Shilpa and Souza, 2020). Clinical evidence from human studies has shown that no side effects or serious complications have been reported when *C. limon* juice or essential oil is used (Avello et al., 2014; Rambod et al., 2020; Kawakami et al., 2021). |
| 5 | *Camellia sinensis* (L.) Kuntze | ○ | ─ | ○ | The meta-analysis showed that consumption of *C. sinensis* polyphenols at a low dose (<582.8 mg/d) induced vasodilation, resulting in a significant reduction of SBP and DBP by 1.98/1.92 mmHg over 12 weeks (Peng et al., 2014; Rawat et al., 2016). Several systematic reviews have confirmed the safety and efficacy of *C. sinensis* tea consumption in patients with hypertension, with no reported side effects (Hu et al., 2018; Mahdavi-Roshan et al., 2020; Xu et al., 2020). |
| 6 | *Zingiber officinale* Rosc. | ○ | ─ | ○ | According to a meta-analysis, *Z. officinale* supplementation in high doses (>3 g/day) and for short intervention periods (≤ 8 weeks) can significantly reduce both SBP and DBP by 6.36 mmHg and 2.12 mmHg, respectively (Sane et al., 2018; Hasani et al., 2019). Previous clinical trials have demonstrated that *Z. officinale* consumption can alleviate mild side effects such as nausea and heartburn, without causing any severe adverse effects (Sane et al., 2018; Nikkhah Bodagh et al., 2019; Anh et al., 2020). |
| 7 | *Petroselinum crispum* (Mill.) Nyman | ○ | ─ | ○ | Administration of the aqueous extract of *P. crispum* reduced systolic, diastolic, and mean arterial BP by inducing vasodilation through an endothelium-independent pathway (Ajebli and Eddouks, 2019). Thus, *P. crispum* is a natural, safe remedy with antihypertensive effects (BP lowering), as no adverse effects were reported after ingesting therapeutic doses were reported. However, a rare case of allergic reactions, such as photodermatosis, was observed (Punoševac et al., 2021). |
| 8 | *Nigella sativa* L. | ○ | ─ | ○ | *N. sativa* seed oil contains several pharmacologically active compounds such as thymol, thymoquinone, thymohydroquinone, dithymoquinone, and α-hederin, which have the potential to lower BP through multiple mechanisms. Research has shown significant reductions in both systolic and diastolic BP after 8 weeks of daily consumption of 5 ml, with observed mean reductions of 8.37/8.54 mmHg (Shoaei‐Hagh et al., 2021; Verma et al., 2021). *N. sativa* is an effective and safe complementary treatment for HTN. Recent scientific research has shown that *N. sativa* can effectively cure HTN and lower BP without causing any harmful side effects (Leong et al., 2013; Musharraf and Arman, 2018). Moreover, no serious adverse effects were observed from using *N. sativa* seeds oil, yet minor adverse effects, such as stomach upset, vomiting, and constipation were found (Shoaei‐Hagh et al., 2021). |
| 9 | *Centella asiatica* L. | ○ | ○ | ○ | Consumption of *C. asiatica* tea has demonstrated efficacy in lowering BP without any observed signs of toxicity or mortality (Astutik et al., 2021; Bunaim et al., 2021). Additionally, a RCT conducted on HTN patients revealed no reported side effects of *C. asiatica*. However, due to the limited sample size of the previous study, further investigation is necessary to validate these findings (Cesarone et al., 2001). |
| 10 | *Cucumis sativus* L. | ○ | ─ | ○ | Several experimental studies have shown that taking 210-400 g of *C. sativus* juice can effectively lower BP in HTN patients without inducing any side effects (Hariyanti et al., 2020; Wibowo and Anita, 2021; Evania et al., 2022). |
| 11 | *Beta vulgaris* L. | ○ | ─ | ○ | Multiple clinical trials and a meta-analysis have demonstrated that consumption of *B. vulgaris* juice significantly reduces SBP and DBP in hypertensive patients by 3.55 mmHg and 1.32 mmHg, respectively, with no significant adverse effects (Bahadoran et al., 2017; Bonilla Ocampo et al., 2018; Arma and Sumarni, 2020; Mirmiran et al., 2020; Benjamim et al., 2022). Additionally, concentrated *B. vulgaris* consumption in DM patients did not result in significant side effects (Karimzadeh et al., 2022). |
| *HTN: hypertension; DM: diabetes mellitus; HM: herbal medicine; BP: blood pressure; RCT: randomized controlled trial  ^a^ ─: effective evidence not available; ○: effectiveness evidence available (either human or animal evidence)  ^b^ ✕: contraindicated for use; △: should be used with caution; ─: safety evidence not available; ○: safe to use | | | | | |
