# Supplementary material for: Safety classification of herbal medicine use among hypertensive patients: a systematic review and meta-analysis
Source: Front Pharmacol. 2024 May 31;15:1321523. doi: 10.3389/fphar.2024.1321523 (PMC11176523; doi:10.3389/fphar.2024.1321523)
Supplement: Supplementary file 6 [file Table4.docx]

Supplementary Table S4. Risk of bias assessments for included studies

| No. | Study  (author, year) | External validity | | | | | Internal validity | | | | | Summary* | | | |
| --- | --- | --- | --- | --- | --- | --- | --- | --- | --- | --- | --- | --- | --- | --- | --- |
|  |  | 1 | 2 | 3 | 4 | 5 | 6 | 7 | 8 | 9 | 10 | Overall  Score | | | |
|  |  | Target  population | Sampling  frame | Sample  selection | Non-  response  bias | Direct  collection of data | Case  definition | Instrument  validity | Mode  of data  collection | Prevalence period | Parameter  of interest |  |  |  |  |
| 1 | Ziyyat et al. (1997) | 1 | 1 | 1 | 1 | 0 | 1 | 1 | 0 | 1 | 0 | 7 | High | |  |
| 2 | Rogers et al. (2001) | 1 | 1 | 1 | 1 | 0 | 1 | 1 | 0 | 0 | 1 | 7 | High | |  |
| 3 | Shafiq et al. (2003) | 1 | 1 | 1 | 1 | 0 | 0 | 1 | 0 | 1 | 0 | 6 | Moderate | |  |
| 4 | Mahfudz & Chan (2005) | 1 | 1 | 0 | 1 | 0 | 0 | 0 | 0 | 1 | 0 | 4 | Moderate | |  |
| 5 | Yeh et al. (2006) | 1 | 1 | 0 | 0 | 0 | 0 | 0 | 1 | 0 | 0 | 3 | Low | |  |
| 6 | Amira & Okubadejo (2007) | 1 | 1 | 1 | 1 | 0 | 0 | 0 | 0 | 1 | 1 | 6 | Moderate | |  |
| 7 | Clement et al. (2007) | 1 | 0 | 1 | 1 | 0 | 1 | 0 | 0 | 1 | 0 | 5 | Moderate | |  |
| 8 | Yilmaz et al. (2007) | 1 | 1 | 1 | 1 | 0 | 1 | 0 | 1 | 0 | 1 | 7 | High | |  |
| 9 | Gohar et al. (2008) | 1 | 1 | 1 | 0 | 0 | 0 | 1 | 0 | 0 | 0 | 4 | Moderate | |  |
| 10 | Olisa & Oyelola (2009) | 1 | 1 | 0 | 0 | 0 | 0 | 0 | 0 | 1 | 0 | 3 | Low | |  |
| 11 | Al-Hamdan et al. (2010) | 1 | 0 | 0 | 0 | 0 | 1 | 0 | 1 | 1 | 1 | 5 | Moderate | |  |
| 12 | Delgoda et al. (2010) | 1 | 1 | 0 | 0 | 0 | 1 | 1 | 0 | 1 | 0 | 5 | Moderate | |  |
| 13 | Nur (2010) | 1 | 1 | 0 | 0 | 0 | 1 | 0 | 1 | 1 | 0 | 5 | Moderate | |  |
| 14 | Osamor et al. (2010) | 1 | 1 | 1 | 1 | 0 | 1 | 1 | 1 | 1 | 0 | 8 | High | |  |
| 15 | Ali-Shtayeh et al. (2013) | 1 | 1 | 0 | 0 | 0 | 0 | 0 | 0 | 0 | 0 | 2 | Low | |  |
| 16 | Bahar et al. (2013) | 1 | 1 | 1 | 1 | 0 | 0 | 0 | 0 | 1 | 0 | 5 | Moderate | |  |
| 17 | Hughes et al. (2013) | 1 | 1 | 1 | 1 | 0 | 1 | 1 | 0 | 0 | 1 | 7 | High | |  |
| 18 | Wazaify et al. (2013) | 1 | 1 | 0 | 0 | 1 | 0 | 0 | 0 | 1 | 0 | 4 | Moderate | |  |
| 19 | Nuwaha et al. (2013) | 1 | 1 | 1 | 0 | 0 | 1 | 1 | 1 | 0 | 0 | 6 | Moderate | |  |
| 20 | Hu et al. (2013) | 1 | 1 | 1 | 1 | 0 | 0 | 1 | 0 | 0 | 0 | 5 | Moderate | |  |
| 21 | Mollaoğlu et al. (2013) | 1 | 1 | 1 | 1 | 0 | 0 | 1 | 0 | 1 | 0 | 6 | Moderate | |  |
| 22 | Açıkgöz et al. (2014) | 1 | 1 | 1 | 0 | 0 | 0 | 0 | 0 | 0 | 0 | 3 | Low | |  |
| 23 | Kretchy et al. (2014) | 1 | 1 | 0 | 0 | 0 | 0 | 0 | 0 | 1 | 0 | 3 | Low | |  |
| 24 | Boima et al. (2015) | 1 | 1 | 1 | 1 | 0 | 1 | 0 | 0 | 1 | 0 | 6 | Moderate | |  |
| 25 | Hughes et al. (2015) | 1 | 1 | 1 | 1 | 0 | 1 | 1 | 0 | 1 | 0 | 7 | High | |  |
| 26 | Li et al. (2015) | 1 | 1 | 1 | 1 | 0 | 1 | 0 | 0 | 0 | 0 | 5 | Moderate | |  |
| 27 | Tajadini et al. (2015) | 1 | 1 | 1 | 0 | 0 | 1 | 0 | 0 | 0 | 0 | 4 | Moderate | |  |
| 28 | Nunes et al. (2015) | 1 | 1 | 1 | 1 | 0 | 1 | 1 | 0 | 1 | 1 | 8 | High | |  |
| 29 | Asfaw et al. (2016) | 1 | 1 | 1 | 0 | 0 | 0 | 0 | 0 | 1 | 0 | 4 | Moderate | |  |
| 30 | Lulebo et al. (2017) | 1 | 1 | 1 | 1 | 0 | 0 | 0 | 0 | 0 | 1 | 5 | Moderate | |  |
| 31 | Marais et al. (2017) | 1 | 1 | 1 | 1 | 0 | 1 | 1 | 1 | 1 | 1 | 9 | High | |  |
| 32 | Baran et al. (2017) | 1 | 1 | 1 | 0 | 0 | 0 | 1 | 0 | 0 | 1 | 5 | Moderate | |  |
| 33 | Liwa et al. (2017) | 1 | 1 | 1 | 0 | 0 | 1 | 1 | 0 | 1 | 0 | 6 | Moderate | |  |
| 34 | Adidja et al. (2018) | 1 | 1 | 0 | 1 | 0 | 1 | 1 | 0 | 1 | 0 | 6 | Moderate | |  |
| 35 | James et al. (2018) | 1 | 1 | 0 | 0 | 0 | 0 | 0 | 0 | 0 | 0 | 2 | Low | |  |
| 36 | Alghamdi et al. (2018) | 1 | 1 | 0 | 1 | 0 | 1 | 1 | 1 | 1 | 0 | 7 | High | |  |
| 37 | Hassaïne et al. (2019) | 1 | 1 | 1 | 1 | 0 | 1 | 0 | 0 | 1 | 1 | 7 | High | |  |
| 38 | Kanjanahattakij et al. (2019) | 1 | 1 | 0 | 1 | 0 | 1 | 1 | 1 | 1 | 1 | 8 | High | |  |
| 39 | Peltzer & Pengpid (2019) | 1 | 1 | 0 | 0 | 0 | 0 | 0 | 0 | 0 | 1 | 3 | Low | |  |
| 40 | Sabery et al. (2019) | 1 | 1 | 0 | 0 | 0 | 1 | 0 | 1 | 1 | 0 | 5 | Moderate | |  |
| 41 | Al-Hadid et al. (2020) | 1 | 1 | 1 | 0 | 0 | 1 | 0 | 0 | 1 | 0 | 5 | Moderate | |  |
| 42 | Alshabi (2020) | 1 | 1 | 0 | 1 | 0 | 1 | 0 | 1 | 1 | 0 | 6 | Moderate | |  |
| 43 | El-Dahiyat et al. (2020) | 1 | 1 | 1 | 0 | 1 | 1 | 0 | 0 | 1 | 0 | 6 | Moderate | |  |
| 44 | Owusu et al. (2020) | 1 | 1 | 1 | 0 | 0 | 0 | 0 | 0 | 1 | 0 | 4 | Moderate | |  |
| 45 | Adeniyi et al. (2021) | 1 | 1 | 1 | 1 | 0 | 1 | 0 | 1 | 0 | 0 | 6 | Moderate | |  |
| 46 | Joachimdass et al. (2021) | 1 | 1 | 1 | 0 | 0 | 1 | 0 | 0 | 0 | 0 | 4 | Moderate | |  |
| 47 | Kifle et al. (2021) | 1 | 1 | 1 | 0 | 0 | 1 | 0 | 1 | 1 | 0 | 6 | Moderate | |  |
| 48 | Thangsuk et al. (2021) | 1 | 1 | 1 | 0 | 0 | 1 | 1 | 0 | 0 | 0 | 5 | Moderate | |  |
| *items adapted from Hoy et al.  *Summary item on the overall risk of study bias; 0–3: low risk; 4–6: moderate risk; 7–10: high risk  0 = yes or reported; 1 = not applicable or not reported  1. Was the study's target population a close representation of the national population in relation to relevant variables?  2. Was the sampling frame a true or close representation of the target population?  3. Was some form of random selection used to select the sample, OR was a census undertaken?  4. Was the likelihood of nonresponse bias minimal?  5. Were data collected directly from the subjects (as opposed to a proxy)?  6. Was an acceptable case definition used in the study?  7. Was the study instrument that measured the parameter of interest shown to have validity and reliability?  8. Was the same mode of data collection used for all subjects?  9. Was the length of the shortest prevalence period for the parameter of interest appropriate?  10. Were the numerator(s) and denominator(s) for the parameter of interest appropriate? | | | | | | | | | | | | | |  |  |
